# Regenerative Medicine in Chronic Inflammatory Dermatoses: A Scoping Review

**DOI:** 10.7759/cureus.119462

**Published:** 2026-11-11

**Authors:** Sandeep Arora, Gulhima Arora

**Affiliations:** 1 Dermatology, Mehektagul Dermaclinic, New Delhi, IND; 2 Dermatology, Apollo Spectra Hospitals, New Delhi, IND

**Keywords:** chronic inflammatory skin disease, evidence mapping, mesenchymal stem cells (mscs), platelet-rich plasma (prp), regenerative medicine therapies, scoping review, stem cells and exosomes

## Abstract

Background: Chronic inflammatory dermatoses persist despite immunomodulatory and biologic therapy, and none of these treatments directly restores damaged tissue. Regenerative products have been tested in several of them, but the clinical evidence has not been mapped.

Objective: This study aimed to map the clinical evidence on platelet concentrates, cell-based products, exosomes and secretome, and biomaterial-based strategies in chronic inflammatory dermatoses and to identify gaps.

Methods: Following Joanna Briggs Institute (JBI) guidance and the Preferred Reporting Items for Systematic Reviews and Meta-Analyses extension for Scoping Reviews (PRISMA-ScR), we searched PubMed on 10 October 2026 (four modality-specific searches limited to clinical trials, observational studies, meta-analyses, and systematic reviews, English, from 2018) for studies on nine dermatoses, including hidradenitis suppurativa (HS). Records were screened at title and abstract level. For each source, we charted design, condition, modality, sample size, and principal finding from the abstract and summarized them in an evidence map and narrative synthesis; no critical appraisal was done. A supplementary search without publication-type limits addressed HS.

Results: Of 99 unique records, 71 sources were charted (37 systematic reviews or meta-analyses and 34 primary studies, which overlap in the trials they cover). Platelet-rich plasma or fibrin (PRP/PRF) accounted for 41, cell-based products for 18, mixed modalities for eight, and exosome or secretome products for four. Sources were most numerous for vitiligo (15), alopecia (15), systemic sclerosis and morphea (14), and oral lichen planus (9). In vitiligo, pooled analyses reported greater repigmentation when PRP was added to light or laser therapy, although several trials found no significant added benefit. In oral lichen planus, PRP/PRF did not differ significantly from corticosteroids in small trials. Cell-based products showed early efficacy signals in atopic dermatitis and mixed results in systemic sclerosis; exosome and secretome data were early-phase. The filtered search found no studies of HS, scaffolds, skin substitutes, or bioprinting. The supplementary search identified seven clinical HS reports, which are not among the 71 sources.

Conclusions: Regenerative approaches have mainly been tested as adjuncts, in small and heterogeneous studies. Research priorities are standardized reporting of product preparation, adequately powered randomized trials, multi-database searching, and testing of biomaterial-based strategies.

## Introduction and background

Psoriasis, atopic dermatitis, alopecia areata, vitiligo, lichen planus, lichen sclerosus, hidradenitis suppurativa (HS), morphea, and systemic sclerosis (SSc) are chronic inflammatory dermatoses. Their shared problem is inflammation that persists and gradually damages the tissue the skin relies on to repair itself [1-8].

The consequences differ by disease (Figure 1). In atopic dermatitis, type 2 and other immune signals weaken the epidermal barrier, resulting in reduced skin hydration which is more easily irritated and colonized, which in turn sustains inflammation [2]. In psoriasis, interleukin-23 and interleukin-17 signaling drives epidermal overgrowth [1]. In vitiligo, immune activity and oxidative stress destroy melanocytes [4,9], and in alopecia areata, T cells attack hair follicles and halt hair growth [3,10]. In oral lichen planus, T-cell activity and injury to the epithelium result in erosions and other manifestations [5,11], and in lichen sclerosus, chronic inflammation thins and scars the genital skin [6,12]. In HS, follicular inflammation progresses to abscesses, tunnels, and scars [7]. In morphea and SSc, ongoing inflammation and vascular injury activate fibroblasts that deposit excess collagen, resulting in skin sclerosis with underlying fat and soft tissue atrophy [8,13,14].

**Figure 1:**
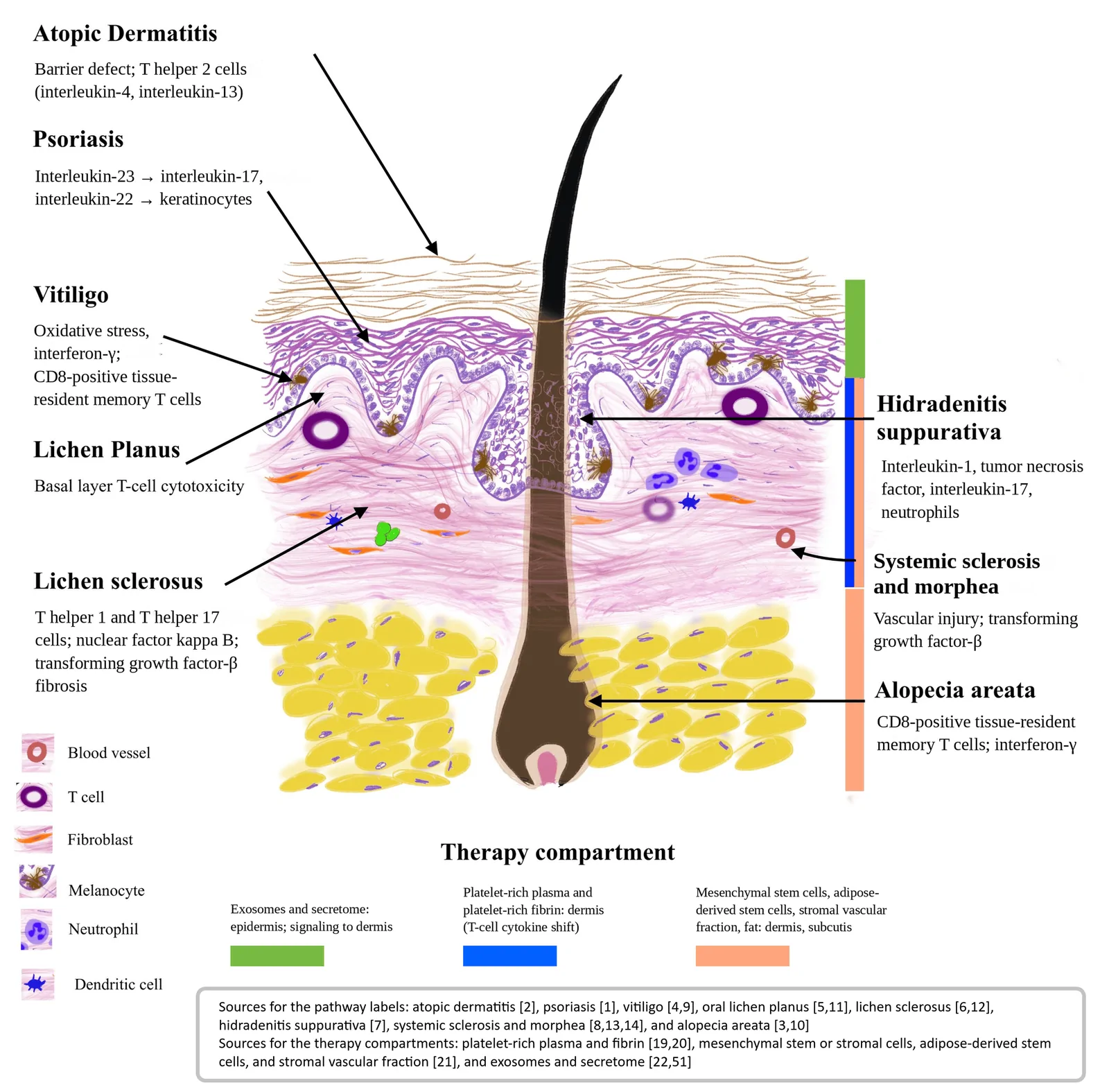
Schematic of the main immune pathways in the conditions reviewed and the skin compartment in which each group of regenerative products is thought to act Pathway labels summarize established disease biology drawn from published reviews and are not findings of this review, and the schematic does not show comparative efficacy. CD8: cluster of differentiation 8 Image Credit: Original drawing created by the authors using Procreate for iPad (Savage Interactive Pty Ltd, Hobart, Australia); no part is reproduced or adapted from another source.

Although treatments have improved, gaps remain. Some patients with moderate-to-severe atopic dermatitis fail to achieve adequate control [15], vitiligo remains difficult to treat [16], morphea has no satisfactory therapy for skin sclerosis and soft tissue atrophy [17], and the first-line corticosteroids used in oral lichen planus carry side effects [18]. Immunomodulatory and biologic drugs suppress inflammation but do not by themselves restore tissue that is already damaged.

Regenerative medicine offers a different treatment paradigm. Instead of only suppressing inflammation, it aims to support repair. In this review, five product groups are covered, defined here as they are used. Platelet concentrates are autologous blood-derived preparations in which concentrated platelets release growth factors on activation; platelet-rich plasma (PRP) is a liquid preparation, and platelet-rich fibrin (PRF), including injectable PRF, is a fibrin-matrix preparation that releases growth factors more gradually [19,20]. Cell-based products comprise mesenchymal stem or stromal cells (MSCs; adipose, bone marrow, or umbilical cord derived, autologous or allogeneic), the stromal vascular fraction (SVF, the heterogeneous cell population obtained by processing adipose tissue), and autologous fat grafting and nanofat (mechanically emulsified fat); their effect is thought to be mainly paracrine and immunomodulatory, not engraftment and differentiation [21]. The secretome is the full set of soluble factors and extracellular vesicles that cells release, including growth factors, cytokines, and microRNA-carrying vesicles. Exosomes are the small-vesicle fraction, one of the components of this secretome [22]. Biomaterials (scaffolds, dermal or skin substitutes, and bioprinted constructs) provide a matrix that supports cells or delivers the other products [20,23]. Platelet concentrates, MSCs, SVF, exosomes and their secretome, and engineered biomaterials are intended to combine immunomodulation with tissue repair [19-23] (Figure 1).

Earlier systematic reviews have mostly examined one product in one condition, for example, PRP in alopecia [24-26], vitiligo [27-30], and oral lichen planus [18,31-33]; fat grafting, SVF, and MSCs in SSc [34-41]; MSCs in atopic dermatitis [15] and psoriasis [42]; and exosomes in alopecia areata [43]. A few have spanned several dermatoses [44-46]. These reviews, which are themselves included in this map, describe small studies with variable preparation methods [47]. Several questions remain unanswered: how the evidence is distributed across diseases and products, which conditions (e.g., HS) have been tested at all, whether newer products such as exosomes and biomaterials have clinical data, and how well product preparation and comparators are reported. A narrative account would not show where the evidence is concentrated, where it is absent, or how these studies were designed. A scoping review, hence, is the appropriate design [48,49].

This scoping review thus reports according to the Preferred Reporting Items for Systematic Reviews and Meta-Analyses extension for Scoping Reviews (PRISMA-ScR) [50] and maps the clinical evidence for regenerative modalities in chronic inflammatory dermatoses. The review question, framed by Population, Concept, and Context, was as follows: What clinical evidence exists on platelet concentrates, cell-based products, exosomes and secretome, and biomaterial-based strategies in people with chronic inflammatory dermatoses, and what are the gaps?

## Review

Methods

Protocol and Reporting

The review followed the Joanna Briggs Institute (JBI) methodological guidance for scoping reviews [49] and the PRISMA-ScR reporting guideline [50]. The protocol was registered retrospectively on the Open Science Framework (OSF) after the PubMed search, screening, and charting were completed (https://osf.io/sj2z8/overview?view_only=376de91dfbe34ea58bc38e1e7d8b4b65); the registration therefore documents the review but was not a prospective protocol. PubMed was the only database searched. The eligibility criteria and search strings were defined before screening began and are reported in full in Appendix A, and the completed checklist is given in Appendix B. Departures from JBI guidance are single-database searching, abstract-level eligibility judgment without full-text retrieval, and second-reviewer verification of a sample only (mentioned in Limitations).

Eligibility Criteria

Population: Eligible participants were people with psoriasis, atopic dermatitis, alopecia areata, vitiligo, lichen planus (cutaneous or oral), lichen sclerosus, HS, morphea, or SSc.

Concept: Eligible interventions were any of four groups of regenerative modalities, namely, platelet concentrates (PRP, PRF, and injectable PRF); cell-based products (MSCs, SVF, nanofat, and autologous fat grafting); exosomes, extracellular vesicles, and secretome or conditioned medium; and biomaterials (scaffolds, skin substitutes, and bioprinted constructs), which can also serve as delivery systems for exosomes [51].

Context: Eligible studies came from clinical dermatology in any setting.** **We included human clinical studies (randomized and non-randomized trials and observational studies) and systematic reviews or meta-analyses published in English from 1 January 2018 to 10 October 2026. Chronic wounds and diabetic foot ulcers were outside the scope of this review. Preclinical studies were not eligible for charting; those retrieved are described separately as context. Sources were classified by the main focus stated in the abstract.

Borderline records: Reviews that also covered conditions or interventions outside the scope above (e.g., androgenetic alopecia, chronic wounds, energy-based devices, or preclinical studies) were charted when the abstract reported clinical data on at least one listed condition and one listed modality; only the eligible data are described, and these reviews are flagged in the charting table. Primary studies with an in vitro or preclinical component were charted when they reported human clinical data. One phase 1 study enrolled healthy volunteers rather than patients [52]; it was kept as a borderline record because it is a clinical study of a product developed for psoriasis, it is flagged, and it is not interpreted as efficacy evidence. Two records that reviewed only preclinical or mechanistic work were not charted and are discussed as context [53,54].

Information Sources and Search

PubMed was searched on 10 October 2026. A common disease block (10 terms covering the nine conditions above, searched in title and abstract) was combined with one of four modality blocks, giving four searches (S1 platelet concentrates, S2 cell-based products, S3 exosomes and secretome, and S4 biomaterials). Each search was limited by date, English language, and publication type (clinical trial, meta-analysis, systematic review, or observational study). The strategies were split because PubMed limits the number of Boolean operators per query. Full strings are in Appendix A. PubMed was the only database searched, and the reference lists of included reviews were not screened. Publication-type limits depend on indexer-assigned labels and exclude untagged records; the consequences for coverage are discussed under Limitations.

Supplementary Search for HS

Filtered searches identified no study of HS; hence, a supplementary PubMed search was run on 27 October 2026 to find HS reports that the publication-type limits would exclude. The term "hidradenitis suppurativa"[tiab] was combined with each of the four modality blocks (S1 to S4; Appendix A), with the same date (1 January 2018 to 10 October 2026) and English-language limits but no publication-type limit; the biomaterial block also included "dermal matrix" and "dermal substitute*". The four searches returned two, five, three, and nine records (19 in total; 17 unique). Titles and abstracts were screened by one reviewer (S.A.), and the selection was checked by the second reviewer (G.A.). Reports of a regenerative product or biomaterial used in people with HS were described narratively and cited in the Results; they were not charted, are not counted in the evidence map, and are not part of the 71 sources.

Selection of Sources of Evidence

We exported titles, abstracts, publication types, and identifiers and de-duplicated records by PubMed identifier. One reviewer (S.A.) screened titles and abstracts against the criteria and recorded a reason for each exclusion. We judged eligibility at the abstract level and did not retrieve full texts at this stage. A random sample of 20 records (14 included and six excluded or context-only; about 20% of the 99 screened records) was set aside for independent verification by a second reviewer (G.A.). The second reviewer's decisions were concordant with the first reviewer's for all 20 records (20/20). The complete screening log is provided as Appendix C.

Data Charting and Synthesis

For each included source, we charted the first author and year, design, condition, modality, population or number of studies, and the principal finding as stated in the Abstract. The complete source-level charting dataset is provided as Appendix D. Where the abstract reported them, we recorded effect estimates with 95% confidence intervals (CIs) and exact p-values. Pooled response proportions (single-group estimates) are distinguished from comparative effects (risk ratios, mean differences, and between-group p-values), and a non-significant difference is reported as such and is not interpreted as evidence of equivalence.

Each source received one primary condition label and one primary modality label (rules also in Appendix D).

Condition: A source on one listed condition was assigned to it; SSc and morphea were combined; sources on alopecia areata and other inflammatory alopecias were combined as alopecia; and a review whose title or abstract covered several dermatoses, or dermatology in general, was labeled "several dermatoses", whereas a review of one listed condition together with conditions outside the list (such as chronic wounds) was assigned to the listed condition.

Modality: A source was assigned to the single modality group named as its intervention of interest; fat grafting, nanofat, SVF, and MSCs were grouped as cell-based; a source naming two or more modality groups as interventions of interest (e.g., fat with PRP) was labeled mixed and counted separately; and a source testing PRP alongside non-regenerative treatments such as light or laser was assigned to PRP.

Reviews and primary studies are tabulated separately because primary studies can appear in more than one review; overlap between reviews was not quantified. Charting was done by one reviewer (S.A.) from the abstracts, and the extracted figures were afterwards re-checked against the PubMed records by the second reviewer (G.A.). No critical appraisal was undertaken, as is permissible in scoping reviews [49,50]. Effect estimates and quality judgments are reported as stated by the original authors, and any statement about the certainty of evidence or risk of bias is attributed to them. Results are summarized by counts, in an evidence map by condition and modality, and in a narrative synthesis.

Results

Selection of Sources of Evidence

The four PubMed searches returned 113 records (S1, 56; S2, 39; S3, 13; S4, 5). After the removal of 14 records retrieved by more than one search, 99 unique records were screened. Twenty-six were excluded: 11 because the condition was outside the scope (e.g., photoaged skin, surgical scars, or pulmonary hypertension), seven because the intervention was not regenerative (e.g., pharmacological agents or hyaluronic acid fillers), seven because the record was non-interventional or a protocol (biomarker studies, a preclinical model review, and a trial protocol), and one because it was published in 2017. Two further records were preclinical or mechanistic reviews and were retained as context for the Discussion. Seventy-one studies were included (Figure 2). Exclusions with reasons are listed in the screening log (Appendix C).

**Figure 2:**
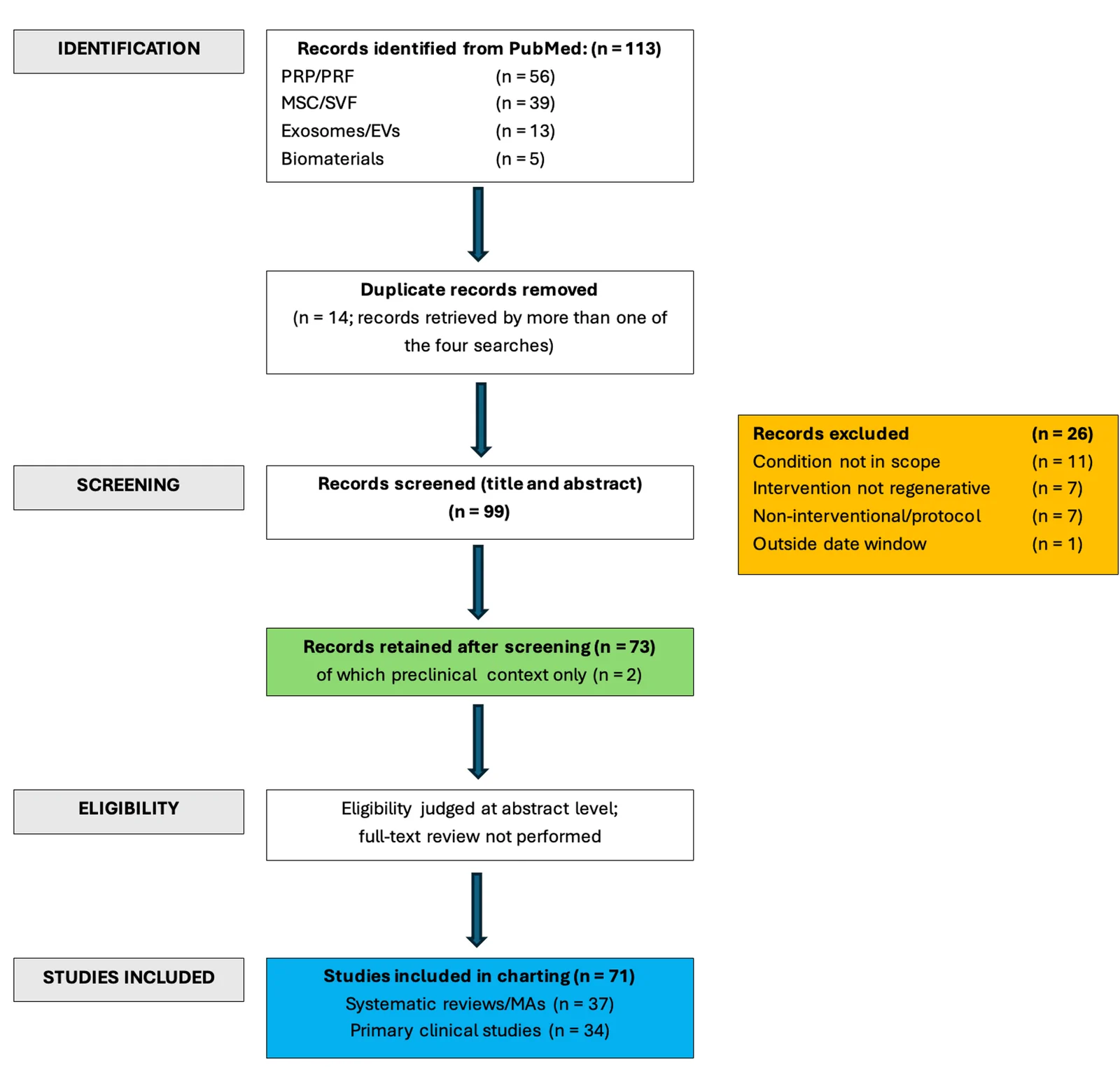
PRISMA-ScR flow diagram of the selection of sources of evidence EVs: extracellular vesicles; MAs: meta-analyses; MSCs: mesenchymal stem or stromal cells; PRF: platelet-rich fibrin; PRISMA-ScR: Preferred Reporting Items for Systematic Reviews and Meta-Analyses extension for Scoping Reviews; PRP: platelet-rich plasma; SVF: stromal vascular fraction Image Credit: Original diagram created by the authors using Microsoft Word (Microsoft Corporation, Redmond, Washington, United States).

Characteristics of the Sources of Evidence

The 71 included studies were published between 2019 and 2026 (by year of the journal issue; one was published online in December 2018), with five in 2026. Thirty-seven were systematic reviews or meta-analyses, one of which was a network meta-analysis, and 34 were primary clinical studies. Most primary studies were randomized or indexed as randomized trials (including pilot, split-mouth, and split-scalp designs); the remainder were non-randomized, single-arm, phase 1, or cohort studies (the design of each study is charted separately). By modality, 41 studies concerned PRP or PRF (21 reviews, 20 primary), 18 cell-based products (nine and nine), four exosome or secretome products (one and three), and eight combined modalities (six and two). No study in the filtered search concerned scaffolds, skin substitutes, or bioprinting. By condition, sources were most numerous for vitiligo and alopecia (15 each; eight and six primary studies), SSc and morphea (14; six primary), and oral lichen planus (9; five primary) (Table 1 and Figure 3B). Because primary studies can appear in several reviews, the 71 sources are not 71 independent pieces of evidence; the 34 primary studies are the better guide to the volume of unique evidence. Individual sources are charted in Table 2.

**Table 1:** Evidence map: number of included studies by condition and modality, with systematic reviews or meta-analyses and primary studies in separate columns

Condition	Modality	Systematic reviews or meta-analyses (n)	Primary studies (n)	Total (n)
Vitiligo	Platelet-rich plasma or fibrin	6	8	14
	Mixed	1	0	1
	All modalities	7	8	15
Alopecia (mainly alopecia areata)	Platelet-rich plasma or fibrin	7	6	13
	Cell-based	1	0	1
	Exosome or secretome	1	0	1
	All modalities	9	6	15
Systemic sclerosis and morphea	Platelet-rich plasma or fibrin	1	0	1
	Cell-based	5	6	11
	Mixed	2	0	2
	All modalities	8	6	14
Oral lichen planus	Platelet-rich plasma or fibrin	4	5	9
	All modalities	4	5	9
Lichen sclerosus	Platelet-rich plasma or fibrin	2	1	3
	Mixed	1	2	3
	All modalities	3	3	6
Psoriasis	Cell-based	2	1	3
	Exosome or secretome	0	2	2
	All modalities	2	3	5
Atopic dermatitis	Cell-based	1	2	3
	Exosome or secretome	0	1	1
	All modalities	1	3	4
Hidradenitis suppurativa	All modalities	0	0	0
Several dermatoses	Platelet-rich plasma or fibrin	1	0	1
	Mixed	2	0	2
	All modalities	3	0	3
All conditions	Platelet-rich plasma or fibrin	21	20	41
	Cell-based	9	9	18
	Exosome or secretome	1	3	4
	Mixed	6	2	8
	Biomaterial (scaffolds, skin substitutes, bioprinting)	0	0	0
	All modalities	37	34	71

**Figure 3:**
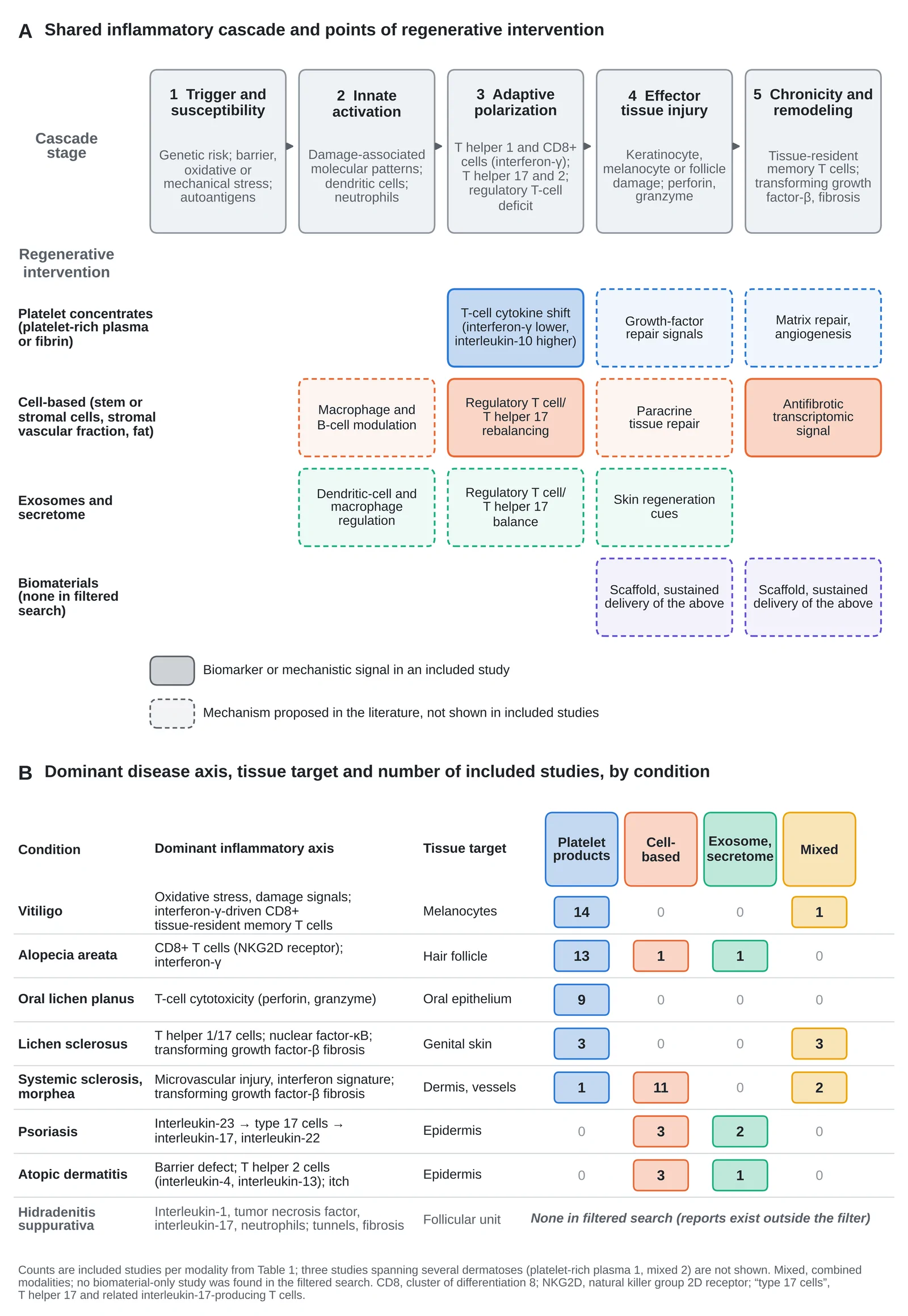
Evidence map and context (A) Shared inflammatory cascade and the points at which each group of regenerative products is thought to act. Solid boxes mark a biomarker or mechanistic signal reported in an included study, and dashed boxes mark mechanisms proposed in the literature but not shown in the included studies. (B) Dominant inflammatory axis, tissue target, and number of included studies by condition and modality (counts as in Table 1). No hidradenitis suppurativa or biomaterial-only study was found by the filtered search. Mechanisms for each product group in panel A are taken from the literature [13-17,27] and the cascade and axis labels from the disease reviews cited in Figure 1. Image Credit: Original artwork created by the authors using Microsoft Word (Microsoft Corporation, Redmond, Washington, United States); no part is reproduced from another source.

**Table 2:** Charting of the included studies (n=71) An asterisk (*) marks records that were charted but are borderline or limited, as described under Methods (Borderline records); the reason for each is given here: no non-PRP control: [16]; out-of-scope condition (androgenetic alopecia): [24,25,66]; out-of-scope condition (primary cicatricial alopecia): [26]; broader intervention scope (energy-based devices): [37]; out-of-scope condition (thumb-base osteoarthritis and other hand conditions): [38]; preclinical component (meta-analysis pooled animal data only): [42]; preclinical or in vitro component: [43,91,97]; multi-condition review: [44]; multi-condition review and preclinical component: [45]; out-of-scope condition (chronic wounds) and borderline condition label (scope stated as medical dermatology): [46]; healthy volunteers: [52]; broader intervention scope: [63,68]; out-of-scope condition (chronic wounds): [64]; out-of-scope condition (androgenetic and cicatricial alopecia): [65]; out-of-scope condition (scarring alopecia only; borderline): [75]; sample size not reported in abstract: [78]; broader intervention scope (mesotherapy): [81]; broader intervention scope (conservative management): [82]; out-of-scope condition (other vulvovaginal disorders): [83]; no abstract and outcomes not extractable: [86]; and descriptive review and no extractable outcome data: [94]. Principal findings are as stated in the abstracts. Effect estimates are shown with 95% CIs and exact p-values when the abstract reported them. Pooled response proportions are single-group estimates, and a non-significant difference is not evidence of equivalence. Judgments about the quality of evidence are those of the original review authors. EASI: Eczema Area and Severity Index; LoSDI: Localized Scleroderma Skin Damage Index; mLoSSI: modified Localized Scleroderma Severity Index; PRF: platelet-rich fibrin; PRP: platelet-rich plasma; SMD: standardized mean difference; SVF: stromal vascular fraction; REU: reticulation/erythema/ulceration

Reference	Condition	Modality	Design	Studies included	Participants	Principal finding (as stated in the abstract)
[15]	Atopic dermatitis	Cell-based	Systematic review and meta-analysis (4 of 5 trials randomized)	5	236	Pooled EASI-50 response proportion at week 12 46.76% (95% CI 32.36 to 61.72) and pooled EASI-75 proportion at week 16 23.97% (95% CI 16.48 to 33.50); single-group response proportions, not comparisons with placebo. Treatment-emergent adverse events 26.86% (95% CI 19.56 to 35.68)
[16]	Vitiligo	PRP or PRF	Randomized controlled trial	Not applicable	40	Erbium:YAG laser plus PRP and microneedling plus PRP both repigmented (20 per arm; no non-PRP control); side effects 5% versus 25%*
[17]	Systemic sclerosis or morphea	Cell-based	Non-randomized controlled trial	Not applicable	44	Sequential fat grafting with SVF gel versus fat grafting alone (morphea): 12-month decrease in LoSDI 4.3 versus 2.1 (p<0.001); decrease in mLoSSI 1.6 versus 0.9 (p=0.13); histologic and transcriptomic antifibrotic signals
[18]	Oral lichen planus	PRP or PRF	Systematic review and meta-analysis	6	109	Platelet concentrates versus topical steroids: pain SMD 0.17 (95% CI -0.47 to 0.81), no significant difference; Thongprasom score SMD -2.88 (95% CI -5.51 to -0.25), described by the authors as not significant although the interval excludes zero
[24]	Alopecia	PRP or PRF	Systematic review and meta-analysis	27	1,117	Controlled trials (18 in androgenetic alopecia, 9 in alopecia areata): hair density mean difference 25.6 hairs/cm^2^ (95% CI 2.62 to 48.57) versus saline in androgenetic alopecia, rated low quality by the authors; alopecia areata evidence (PRP versus triamcinolone) rated very low quality*
[25]	Alopecia	PRP or PRF	Systematic review	19	460	Articles covered alopecia areata (3 studies, 71 patients) and androgenetic alopecia (16 studies, 389 patients); most studies reported hair growth; heterogeneity prevented pooling*
[26]	Alopecia	PRP or PRF	Systematic review	32	621	Participants had alopecia areata (a further 19 had primary cicatricial alopecia); PRP superior as monotherapy in 5 studies and comparable to intralesional steroids in 6 (alopecia areata); alleviated progression in 9 (primary cicatricial alopecia)*
[27]	Vitiligo	PRP or PRF	Network meta-analysis of randomized trials (whole excimer-combination network)	38	1,841	PRP combined with excimer light enhanced 50% or greater repigmentation (risk ratio 1.76; 95% CI 1.32 to 2.35); studies and participants refer to the whole network
[28]	Vitiligo	PRP or PRF	Systematic review and meta-analysis	7	366	PRP plus fractional laser: mean repigmentation grade, mean difference 1.58 (95% CI 1.08 to 2.07; p<0.01); adverse events risk ratio 0.86 (95% CI 0.69 to 1.07; p=0.17)
[29]	Vitiligo	PRP or PRF	Meta-analysis	6	302	PRP plus 308-nm excimer improved response versus phototherapy alone; adverse events reported as comparable
[30]	Vitiligo	PRP or PRF	Systematic review and meta-analysis	11	670	PRP plus excimer or fractional carbon dioxide laser effective; no added benefit reported with narrowband ultraviolet B or noncultured epidermal cell suspension (participants reported as cases)
[31]	Oral lichen planus	PRP or PRF	Systematic review and meta-analysis (6 of 7 articles pooled)	7	141	PRP versus intralesional steroids: pain effect size -0.34 (95% CI -0.94 to 0.27; p=0.274), lesion size effect size -0.10 (95% CI -0.58 to 0.39; p=0.697); no significant differences; participants are those in the six pooled articles
[32]	Oral lichen planus	PRP or PRF	Systematic review of randomized controlled trials	7	Not stated	PRP and injectable PRF improved pain and lesion severity; mostly no significant difference versus corticosteroids
[33]	Oral lichen planus	PRP or PRF	Systematic review	5	Not stated	PRP improved symptoms; efficacy described by the review authors as comparable to corticosteroids
[34]	Systemic sclerosis or morphea	Cell-based	Systematic review and meta-analysis	15	Not stated	Fingertip symptoms improved; skin thickness change not significant; SVF had highest adverse-event proportion
[35]	Systemic sclerosis or morphea	Cell-based	Systematic review	13	Not stated	Systemic sclerosis digital ulcers: healing 66-100% with fat grafting (4 studies) and 32-60% with SVF (3 studies)
[36]	Systemic sclerosis or morphea	Mixed	Systematic review	18	Not stated	Facial scleroderma: most studies showed symptom improvement; no treatment superior; quality weak (15 studies) or moderate (3 studies) as judged by the review authors
[37]	Systemic sclerosis or morphea	Mixed	Systematic review	28	732	15 of the 28 articles concerned energy-based devices; positive outcomes in all but one intralesional-injection study (SVF gel, fat, PRP plus SVF); no life-threatening adverse events*
[38]	Systemic sclerosis or morphea	Cell-based	Systematic review	25	909	Systemic sclerosis accounted for 7 studies; autologous fat grafting improved pain and function in systemic sclerosis and thumb-base osteoarthritis; heterogeneous techniques*
[39]	Systemic sclerosis or morphea	PRP or PRF	Systematic review	Not stated	Not stated	Systemic sclerosis and localized scleroderma: improved mouth handicap, skin elasticity, ulcer healing, and Localized Scleroderma Cutaneous Assessment Tool; adverse events mild and self-limited
[40]	Systemic sclerosis or morphea	Cell-based	Systematic review and meta-analysis	9	133	Effects expressed versus baseline; mesenchymal stem or stromal cell therapy improved skin thickening, lung function, mouth opening, and digital ulcers; no severe adverse events
[41]	Systemic sclerosis or morphea	Cell-based	Systematic review	11	101	Improvements in vascular and pain outcomes in almost all cases; evidence level mostly 4
[42]	Psoriasis	Cell-based	Systematic review and meta-analysis	21	Not stated	5 clinical and 16 preclinical studies; meta-analysis (10 preclinical studies, 343 animals) showed reduced Psoriasis Area and Severity Index and epidermal thickness; clinical data not pooled*
[43]	Alopecia	Exosome or secretome	Systematic review (clinical and experimental evidence)	40	Not stated	Only 2 studies were clinical; density gains 9-31 hairs/cm^2^ in clinical reports; mild transient adverse events; randomized trials lacking*
[44]	Several dermatoses	Mixed	Systematic review	64	2,888	Regenerative methods (PRP, SVF, mesenchymal stem cells, conditioned media, cell transplants) promising across selected dermatoses*
[45]	Several dermatoses	Mixed	Systematic review (preclinical and clinical)	Not stated	Not stated	Psoriasis, atopic dermatitis, localized scleroderma, and acne; adipose-derived mesenchymal stem cells reduced psoriatic features; their exosomes alleviated atopic dermatitis (largely preclinical)*
[46]	Several dermatoses	PRP or PRF	Systematic review	14	Not stated	Articles covered chronic wounds and vitiligo; two studies documented benefit of adjunctive PRP in stable vitiligo*
[52]	Psoriasis	Exosome or secretome	Phase 1 safety study	Not applicable	10	Topical mesenchymal stem cell exosome ointment in healthy volunteers: well tolerated; no serious adverse events; no patients with psoriasis enrolled*
[55]	Vitiligo	PRP or PRF	Randomized controlled trial	Not applicable	60	Latanoprost plus fractional carbon dioxide laser plus PRP gave greater improvement than latanoprost plus laser or latanoprost alone (three arms)
[56]	Vitiligo	PRP or PRF	Comparative study (indexed as randomized controlled trial)	Not applicable	52	PRP plus excimer produced higher response than excimer alone
[57]	Vitiligo	PRP or PRF	Randomized controlled trial	Not applicable	36	Excimer with or without PRP: no significant difference in repigmentation (72 patches)
[58]	Vitiligo	PRP or PRF	Randomized controlled trial	Not applicable	20	All arms improved; no significant difference between fractional carbon dioxide laser, PRP, and narrowband ultraviolet B combinations
[59]	Vitiligo	PRP or PRF	Randomized controlled trial	Not applicable	15	Adding PRP to noncultured epidermal cell suspension gave no significant additional benefit in acral or juxta-articular vitiligo (30 lesions)
[60]	Vitiligo	PRP or PRF	Randomized controlled trial	Not applicable	21	Epidermal cell suspension in PRP versus phosphate-buffered saline (two patches per patient): repigmentation 75.6% versus 65% at 6 months (p=0.0036)
[61]	Vitiligo	PRP or PRF	Randomized controlled trial	Not applicable	60	Narrowband ultraviolet B plus PRP versus plus latanoprost: excellent response 13.3% versus 26.7% (no significant difference in degree of repigmentation); Vitiligo Extent Score for a Target Area favored latanoprost (p=0.032); PRP response described by the authors as more stable
[62]	Vitiligo	Mixed	Systematic review	48	2,186	Regenerative approaches reported effective with no serious adverse events; the review authors judged 48.8% of the included studies at high risk of bias
[63]	Vitiligo	PRP or PRF	Systematic review and meta-analysis	73	2,911	Surgical therapies reviewed, including PRP; benefit reported for ablative laser or microneedling with narrowband ultraviolet B*
[64]	Vitiligo	PRP or PRF	Systematic review and meta-analysis	27	Not stated	Studies covered wounds and vitiligo; PRP gave better repigmentation than controls in vitiligo; wound outcomes outside scope*
[65]	Alopecia	PRP or PRF	Systematic review	61	Not stated	Preliminary results described as promising for androgenetic alopecia, alopecia areata, and cicatricial alopecia; heterogeneous data*
[66]	Alopecia	Cell-based	Systematic review	6	188	All studies reported improved hair density; no severe adverse events*
[67]	Alopecia	PRP or PRF	Systematic review	8	Not stated	PRP plus minoxidil gave highest improvement; alopecia areata efficacy 31.7-76%
[68]	Alopecia	PRP or PRF	Systematic review (PRP, ultraviolet radiation, and laser therapies)	46	Not stated	Of 644 articles screened, 46 were included; strong potential reported but high-quality evidence lacking (authors' statement)*
[69]	Alopecia	PRP or PRF	Randomized controlled trial	Not applicable	60	Intralesional triamcinolone more effective than PRP for complete response (the abstract states both p=0.05 and p<0.05); 30 per arm
[70]	Alopecia	PRP or PRF	Randomized controlled trial (split-scalp)	Not applicable	50	Regrowth greatest with triamcinolone, then PRP, and then placebo (p=0.016 across three arms; no pairwise test in abstract); dermoscopic improvement similar for PRP and triamcinolone (p=0.448)
[71]	Alopecia	PRP or PRF	Randomized controlled trial	Not applicable	376	Number as stated in the abstract. PRP versus 5% minoxidil: mean Severity of Alopecia Tool score 1.48 versus 1.54 at 3 months; no significant difference (p=0.483)
[72]	Alopecia	PRP or PRF	Randomized controlled trial	Not applicable	24	Diphenylcyclopropenone with or without PRP (13 versus 11 patients): regrowth 53.85% versus 54.5%; no significant difference
[73]	Alopecia	PRP or PRF	Randomized controlled trial	Not applicable	27	Severity of Alopecia Tool change not significant versus baseline; interferon-gamma and interleukin-17 messenger RNA fell; interleukin-10 and forkhead box P3 (FOXP3) rose
[74]	Alopecia	PRP or PRF	Randomized controlled trial	Not applicable	60	Injection versus laser-assisted or microneedling-assisted topical PRP (three arms): similar improvement; injection more painful
[75]	Alopecia	PRP or PRF	Systematic review	11	Not stated	Scarring alopecia: PRP described as slowing progression with reduced inflammatory signs; evidence limited to case reports*
[76]	Oral lichen planus	PRP or PRF	Randomized controlled trial (pilot)	Not applicable	20	PRP versus triamcinolone: complete remission 80% versus 70% (not significant)
[77]	Oral lichen planus	PRP or PRF	Randomized controlled trial (split-mouth)	Not applicable	24	Injectable PRF versus corticosteroid: similar reductions in pain and lesion size at 6 months
[78]	Oral lichen planus	PRP or PRF	Randomized controlled trial (split-mouth)	Not applicable	Not reported in abstract	PRF versus triamcinolone: lesion reduction 59.8% versus 59.2%; no significant difference*
[79]	Oral lichen planus	PRP or PRF	Randomized controlled trial (split-mouth)	Not applicable	12	Injectable PRF versus triamcinolone at 4 weeks: visual analog scale reduction 68.5% versus 91% and REU score reduction 74% versus 91%; no significant between-group difference (p>0.05; exact value not given in abstract)
[80]	Oral lichen planus	PRP or PRF	Randomized controlled trial	Not applicable	80	PRP gel plus tacrolimus: higher effective rate, smaller lesion area, lower pain, and lower recurrence than tacrolimus alone
[81]	Lichen sclerosus	Mixed	Systematic review	13	Not stated	Studies of PRP, adipose-derived stem cells, and mesotherapy: improved symptoms, quality of life, sexual function, and tissue quality; small samples, few controls, short follow-up*
[82]	Lichen sclerosus	PRP or PRF	Systematic review	17	Not stated	Male genital lichen sclerosus: steroids remain mainstay; PRP and tacrolimus supported as alternatives or adjuvants*
[83]	Lichen sclerosus	PRP or PRF	Systematic review	18	Not stated	Searched PubMed and Embase to October 2024; PRP injection to the vulvovaginal area as a stand-alone technique; vulvar lichen sclerosus among indications; protocols heterogeneous*
[84]	Lichen sclerosus	Mixed	Randomized controlled trial (pilot)	Not applicable	20	19 assessed. Lipofilling plus PRP versus clobetasol: symptoms, signs, and inflammation improved; no elasticity gain; no related adverse events
[85]	Lichen sclerosus	Mixed	Randomized controlled trial	Not applicable	40	Adipose-derived SVF alone and with PRP both improved at 6 months; combination favored in early disease and discouraged in late disease
[86]	Lichen sclerosus	PRP or PRF	Randomized controlled trial (letter)	Not applicable	Not reported in abstract	Double-blind placebo-controlled trial of intradermal PRP in vulvar lichen sclerosus; no abstract; outcomes not extractable from the PubMed record*
[87]	Systemic sclerosis or morphea	Cell-based	Randomized controlled trial	Not applicable	88	Adipose-derived regenerative cells (n=48) versus placebo (n=40): primary Cochin Hand Function Scale end point not met (mean improvement at 48 weeks 11.0 versus 8.9; p=0.299); trend in diffuse systemic sclerosis (nominal p=0.069); well tolerated
[88]	Systemic sclerosis or morphea	Cell-based	Randomized controlled trial (phase 2)	Not applicable	40	Adipose-derived SVF not superior to placebo for hand function at 3 months
[89]	Systemic sclerosis or morphea	Cell-based	Randomized controlled trial	Not applicable	20	Open label, control medical treatment only; SVF plus fat micrografts: less pain and fewer digital ulcers versus control; no adverse events
[90]	Systemic sclerosis or morphea	Cell-based	Randomized controlled trial (pilot)	Not applicable	18	Localized scleroderma: adipose-derived stem cell-assisted fat grafting, fat retention 49.8% versus 31.8% (SVF) versus 21.9% (conventional)
[91]	Systemic sclerosis or morphea	Cell-based	Open cohort with in vitro study	Not applicable	62	Oro-facial systemic sclerosis: stem cell-enriched lipotransfer improved fibrosis; reduced transforming growth factor-beta 1 and connective tissue growth factor in co-culture*
[92]	Psoriasis	Cell-based	Phase 1/2a single-arm trial	Not applicable	17	Umbilical cord mesenchymal stem cell transplantation safe and partially effective: 8 of 17 (47.1%) had at least 40% Psoriasis Area and Severity Index improvement (2 of 8 men and 6 of 9 women); regulatory T-cell rise and T helper 17 fall in responders
[93]	Psoriasis	Exosome or secretome	Randomized controlled trial (indexed)	Not applicable	12	12 patients for efficacy, 7 for safety; secretome with hyaluronic acid sponge: modified Psoriasis Area and Severity Index reduction up to 33%; plaque size down 41%; no adverse reactions
[94]	Psoriasis	Cell-based	Systematic review (descriptive)	Not stated	Not stated	Describes mesenchymal stem cell interaction with the psoriatic inflammatory microenvironment and therapeutic potential*
[95]	Atopic dermatitis	Cell-based	Randomized controlled trial (phase 2)	Not applicable	114	Autologous adipose-derived mesenchymal stem cells: EASI-75 at week 16 in 23.7% (14/59) versus 7.3% (4/55) with placebo (p=0.016)
[96]	Atopic dermatitis	Cell-based	Phase 1/2 trial	Not applicable	92	Phase 1 n=20 and phase 2 n=72; clonal bone marrow-derived mesenchymal stem cells: higher EASI-50 response at week 12 versus placebo (p=0.038); Dermatology Life Quality Index and pruritus differences not significant
[97]	Atopic dermatitis	Exosome or secretome	Clinical and in vitro study	Not applicable	Not stated	Mild atopic dermatitis; conditioned medium applied for 4 weeks improved skin barrier (corneometry up, transepidermal water loss down)*

Results by Condition

Vitiligo: Fifteen sources, all but one on PRP, addressed vitiligo, and PRP was almost always tested as an adjunct to light, laser, or surgery. In a network meta-analysis of 38 randomized trials of excimer-light combinations (1,841 participants in the whole network), adding PRP to excimer light increased 50% or greater repigmentation (risk ratio 1.76; 95% CI 1.32 to 2.35) [27]. A meta-analysis of seven randomized trials (366 patients) reported a greater repigmentation grade with PRP plus fractional laser (mean difference 1.58; 95% CI 1.08 to 2.07; p<0.01) [28], and a further meta-analysis of six studies (302 patients) supported PRP with 308-nm excimer therapy [29]. Another meta-analysis found PRP effective with excimer or fractional carbon dioxide laser but reported no added benefit with narrowband ultraviolet B or noncultured epidermal cell suspension [30]. Primary trials were less uniform. A randomized trial comparing fractional Er:YAG laser with microneedling as PRP delivery methods (40 patients, no non-PRP control) reported repigmentation in both groups [16], PRP added to latanoprost and fractional laser improved outcomes [55], and PRP with excimer laser outperformed excimer alone in one trial [56]. Other randomized trials found no significant added benefit from PRP with excimer light [57], with fractional laser and narrowband ultraviolet B [58], or with epidermal cell suspension in acral vitiligo [59], while a trial in stable vitiligo reported greater repigmentation when the cell suspension was prepared in PRP (75.6% versus 65%; p=0.0036) [60]. In a comparison of latanoprost with PRP, each added to narrowband ultraviolet B (60 patients), the excellent-response rate was numerically lower with PRP (13.3% versus 26.7%; no significant difference in degree of repigmentation), the Vitiligo Extent Score for a Target Area favored latanoprost (p=0.032), and the authors reported a more stable response with PRP [61]. A broader review of 48 articles (2,186 patients) concluded that regenerative approaches were effective and safe, and its authors judged 48.8% of the included studies to be at high risk of bias [62]. Two further reviews included PRP among broader interventions: a review of surgical therapies in vitiligo (73 studies, 2,911 patients), which found benefit for ablative laser or microneedling combined with narrowband ultraviolet B [63], and a meta-analysis of PRP in chronic wounds and vitiligo (27 studies) in which PRP gave better repigmentation than controls in vitiligo [64].

Alopecia: Fifteen sources addressed alopecia, mainly alopecia areata. Several of the reviews [24,25,65,66] also covered androgenetic alopecia, which lies outside the listed conditions; those data are not summarized here except where stated. A meta-analysis of 27 controlled trials (18 in androgenetic alopecia and nine in alopecia areata) reported, in androgenetic alopecia, a pooled hair-density gain against saline (mean difference 25.6 hairs/cm²; 95% CI 2.62 to 48.57), which its authors rated as low-quality evidence; they rated the evidence for PRP versus triamcinolone in alopecia areata as very low quality [24]. A review of 32 studies (621 patients with alopecia areata) reported PRP superior as monotherapy in five studies and comparable to intralesional corticosteroids in six [26]. Other reviews reported a majority of favorable outcomes but substantial heterogeneity [25,65,67], and a review of procedural therapies for alopecia areata stated that high-quality evidence was lacking [68]. In two randomized trials that compared PRP with intralesional triamcinolone, neither of which was designed as a non-inferiority trial, triamcinolone gave the better result. In an open-label trial of 60 patients, triamcinolone produced more complete responses than PRP (the abstract states both p=0.05 and p<0.05) [69]. In a split-scalp trial of 50 patients, regrowth was greatest with triamcinolone, then PRP, and then placebo (p=0.016 across the three treatments, with no pairwise test reported in the abstract), whereas dermatoscopic improvement was similar with PRP and triamcinolone (p=0.448) [70]. PRP did not differ significantly from 5% minoxidil (mean Severity of Alopecia Tool score 1.48 versus 1.54 at three months; p=0.483) in a trial reporting 376 patients in its abstract [71], adding PRP to diphenylcyclopropenone gave no significant benefit [72], and a placebo-controlled trial found limited efficacy, with a shift towards a more regulatory cytokine profile (lower interferon-gamma and interleukin-17 and higher interleukin-10 and FOXP3 expression) [73]. Delivery by laser or microneedling-assisted topical PRP gave similar improvement to injection with less pain [74]. For scarring alopecia, a review of 11 studies found evidence limited to case reports [75]. SVF was assessed in nonscarring alopecia (androgenetic and areata) in a review of six studies (188 patients), all of which reported improved hair density [66], and a review of exosome-based therapy for alopecia areata (40 studies, clinical and experimental) reported density gains of 9-31 hairs/cm² in clinical reports but no randomized trials [43].

Oral lichen planus: All nine sources on oral lichen planus used PRP, PRF, or injectable PRF, and the comparator was usually a corticosteroid. In a meta-analysis of six randomized trials (109 patients), pain did not differ significantly between platelet concentrates and topical steroids (standardized mean difference 0.17; 95% CI −0.47 to 0.81); for the Thongprasom score, the estimate was −2.88 (95% CI −5.51 to −0.25), which the authors described as not significant [18]. Another meta-analysis (six of seven articles pooled; 141 participants) found no significant difference between intralesional PRP and intralesional steroid in pain (effect size −0.34; 95% CI −0.94 to 0.27; p=0.274) or lesion size (effect size −0.10; 95% CI −0.58 to 0.39; p=0.697), and its authors reported substantial heterogeneity and publication bias [31]. Two further reviews reported improvement with PRP or injectable PRF and mostly no significant difference from corticosteroids [32,33]. Primary randomized trials were small. Two split-mouth trials and one pilot trial found no significant difference from corticosteroids [76-78], and in another split-mouth trial (12 patients), the visual analog scale reduction was numerically larger with triamcinolone than with injectable PRF (91% versus 68.5%; difference reported as not significant) [79]. One trial of 80 patients reported a higher response rate and lower recurrence when PRP gel was added to tacrolimus ointment than with tacrolimus alone [80]. Because these trials were small, a non-significant difference should not be read as equivalence.

Lichen sclerosus: Six sources addressed lichen sclerosus, three of them reviews. A review of 13 studies on PRP, adipose-derived stem cells, and mesotherapy in vulvar disease reported improved symptoms, quality of life, and sexual function, limited by small samples, absent control groups, and short follow-up [81]. A review of 17 studies on conservative management of male genital disease supported PRP as an alternative or adjuvant after topical corticosteroids [82]. A review of PRP in vulvovaginal disorders (18 studies) listed lichen sclerosus among the indications and found highly variable protocols [83]. In a pilot randomized trial, lipofilling with PRP reduced symptoms and inflammatory infiltrate compared with clobetasol, without an improvement in vulvar elasticity [84]. In a trial of 40 patients, SVF and SVF with PRP both improved genital disease at six months, and the combination was favored in early disease and discouraged in late disease [85]. A double-blind, placebo-controlled trial of PRP in vulvar disease was identified, but its PubMed record has no abstract, and its results could not be extracted [86].

SSc and morphea: Fourteen sources addressed SSc or morphea, and this was the area where cell-based products were most studied. Reviews of autologous fat, SVF, and adipose-derived stem cells in SSc (15 studies) reported improved fingertip symptoms, with no significant change in skin thickness and the highest proportion of adverse events with SVF [34]. Other reviews covered digital ulcers (13 studies, with healing of 66-100% after fat grafting and 32-60% after SVF) [35], facial disease (18 studies, none judged superior, with quality rated weak or moderate by the review authors) [36], procedures in morphea and SSc (28 articles, 732 patients) [37], autologous fat grafting for hand conditions (25 studies, with the strongest evidence in SSc and thumb-base osteoarthritis) [38], and PRP [39]. A meta-analysis of MSC therapy (nine studies, 133 patients, with effects expressed against baseline) reported improved skin thickening, lung function, mouth opening, and digital ulcers without severe adverse events, but no significant change in the Raynaud or Cochin hand function scores at six months [40], whereas a systematic review of human MSC use was limited to case series and reports (101 patients) [41]. Primary randomized evidence was mixed. A trial of adipose-derived regenerative cells (88 patients) did not meet its primary hand-function end point (mean improvement in the Cochin Hand Function Scale at 48 weeks 11.0 versus 8.9; p=0.299), although trends were seen in diffuse disease [87], and a multicenter phase 2 trial found no superiority of SVF over placebo [88]. A smaller trial of SVF with fat micrografts reported less pain and fewer digital ulcers [89]. In localized scleroderma, expanded adipose-derived stem cells improved fat retention in a pilot trial of 18 patients [90], and in a non-randomized trial of 44 patients with morphea, sequential fat grafting with SVF gel improved the Localized Scleroderma Skin Damage Index (LoSDI; decrease 4.3 versus 2.1; p<0.001) but not the modified Localized Scleroderma Severity Index (mLoSSI; decrease 1.6 versus 0.9; p=0.13) compared with fat grafting alone [17]. An open cohort of 62 patients reported benefit from stem cell-enriched lipotransfer in oro-facial SSc [91].

Psoriasis and atopic dermatitis: Evidence in these two conditions came almost entirely from cell-based and exosome or secretome products and was early-phase. For psoriasis, a phase 1/2a single-arm trial of umbilical cord MSCs reported safety and partial efficacy, with at least 40% improvement in the Psoriasis Area and Severity Index in eight of 17 patients (two of eight men and six of nine women), a rise in regulatory T cells, and a fall in Th17 cells in responders [92]. A topical secretome and hyaluronic acid sponge reduced the modified Psoriasis Area and Severity Index by up to 33% in 12 patients [93], and a phase 1 study in 10 healthy volunteers (not patients with psoriasis) found a topical MSC-exosome ointment well tolerated [52]. A meta-analysis of MSC transplantation in psoriasis pooled only preclinical data (10 studies, 343 animals) and identified five clinical studies [42], and a descriptive review addressed the interaction between MSCs and the psoriatic microenvironment [94]. For atopic dermatitis, a meta-analysis of five clinical trials (236 participants) estimated a pooled 50% improvement rate in the Eczema Area and Severity Index (EASI-50) of 46.76% (95% CI 32.36 to 61.72) at week 12 and a pooled EASI-75 rate of 23.97% (95% CI 16.48 to 33.50) at week 16; these are single-group response proportions, not comparisons with placebo. Treatment-emergent adverse events occurred in 26.86% (95% CI 19.56 to 35.68) [15]. A multicenter phase 2 trial of autologous adipose-derived MSCs (114 participants) reported EASI-75 at week 16 in 23.7% (14/59) versus 7.3% (4/55) with placebo (p=0.016) [95], and a phase 1/2 trial of clonal bone marrow-derived MSCs reported a higher EASI-50 response than placebo at week 12 (p=0.038), without significant differences in Dermatology Life Quality Index or pruritus scores [96]. A small study of umbilical cord blood MSC conditioned medium reported improved skin barrier measures in mild disease, but most of its data were in vitro [97].

Reviews spanning several dermatoses: Three reviews spanned several dermatoses: a review of 64 studies (2,888 patients) of regenerative methods in specific dermatologic disorders [44], a review of adipose-derived MSCs and their derivatives in psoriasis, atopic dermatitis, localized scleroderma, and acne, which drew largely on preclinical data [45], and a review of PRP in medical dermatology that documented benefit as an adjunct in stable vitiligo [46].

Supplementary search for HS (reports not part of the 71 mapped sources): No HS study was identified by the filtered search. This reflects the publication-type filter, which excludes case reports and many retrospective studies, and not an absence of HS literature. The supplementary search without publication-type limits (see Methods) retrieved 17 unique records. These reports are not part of the 71 mapped sources, Table 1 or Figure 3. Seven were clinical reports of a regenerative product or biomaterial in people with HS. One was a comparative retrospective study of 61 patients [98]: PRP-injected local skin flaps were compared with flaps without PRP and showed a trend toward better healing and fewer complications, and, separately, an acellular dermal matrix co-grafted with a split-thickness skin graft gave better outcomes than skin graft alone. One was a pilot case series of six participants (eight defects) in whom an extracellular matrix graft was used after excision of Hurley stage III axillary disease, with complete healing in all defects [99]. Five were case reports: acellular dermal substitute reconstruction of axillary HS [100], an acellular dermal matrix and split-thickness skin graft co-graft in two patients [101], cryopreserved human allograft used as a bridge to skin grafting [102], a mini skin graft combined with PRF in a child [103], and local injection of autologous adipose tissue [104]. Three further records were preclinical or ex vivo studies of mesenchymal cells in HS (e.g., [105,106]), and seven were not eligible (e.g., an unrelated disease or a device review in which HS was one of the several applications). Clinical evidence in HS consists mainly of case reports and small series, but it is not absent.

Results by Modality and Safety

Exosome and secretome products were represented by one review and three primary studies, of which only one reported efficacy in patients with a dermatosis [93]; the others were a healthy-volunteer safety study [52] and a study with mostly in vitro data [97]. The search for scaffolds, biomaterials, skin substitutes, and bioprinting returned five records; four were not eligible, and one (the facial scleroderma review [36]) was also retrieved by another search and is charted under mixed modalities. Most sources reported mild, self-limited, or no serious adverse events [39,40,52,96], although the studies were small and short, and in SSc, the highest proportion of adverse events was associated with SVF [34].

Mechanistic Outcomes

Few included studies reported mechanistic outcomes, and these were exploratory: lower interferon-gamma and interleukin-17 and higher interleukin-10 and FOXP3 expression after PRP in alopecia areata [73], a rise in regulatory T cells and a fall in Th17 cells and serum interleukin-17 after umbilical cord MSCs in psoriasis [92], and transcriptomic antifibrotic signatures after fat and SVF gel grafting in morphea [17].

Discussion

Summary and Interpretation of the Evidence

This scoping review mapped 71 sources (37 reviews and 34 primary studies) on regenerative approaches in chronic inflammatory dermatoses. The map shows an uneven field. Sources are most numerous for vitiligo, alopecia, oral lichen planus, and SSc and fewer for psoriasis, atopic dermatitis, and lichen sclerosus, and the filtered search found none for HS or biomaterial-based strategies. Because reviews overlap with the primary studies they pool, the counts overstate the amount of independent evidence.

The dominant pattern is the use of PRP as an adjunct to other treatment or as an alternative to corticosteroids. In vitiligo, pooled analyses reported greater repigmentation when PRP was added to excimer or fractional laser [27-29], although several individual trials found no significant added benefit [57-59]. In oral lichen planus, meta-analyses and trials found no significant difference between PRP or PRF and corticosteroids [18,31,32]; because most trials were small, this should not be read as equivalence. In alopecia areata, the review authors rated the evidence as low or very low quality [24], and in two head-to-head trials, intralesional triamcinolone gave greater regrowth than PRP [69,70]; neither trial was designed to test non-inferiority, so PRP cannot be called non-inferior on this evidence.

Cell-based products show a different profile. The pooled EASI-50 proportion for MSCs in atopic dermatitis [15] is not a comparison with placebo and rests on 236 participants, too few to define effect size or durability; one phase 2 trial reported a significant EASI-75 difference over placebo [95]. In SSc, results diverge by outcome and design: reviews report improvement in fingertip symptoms and ulcers [34,35], whereas two randomized trials did not meet their primary hand-function end points [87,88]. Exosome and secretome products have so far been tested in early-phase studies of topical use [52,93], and the systematic review of exosomes in alopecia areata found no randomized trials [43].

Clinical Implications

For clinicians, the mapped evidence suggests that regenerative products are, at present, adjuncts to established treatment. The most frequently reported favorable findings concern PRP combined with light or laser therapy in vitiligo [27-29], and PRP or PRF has been described as a possible alternative to corticosteroids in oral lichen planus [18]. In alopecia areata, neither head-to-head trial showed PRP to be better than intralesional triamcinolone, and triamcinolone gave the greater regrowth in both [69,70]. These trials were not designed to test non-inferiority, so they do not show that PRP is non-inferior; PRP is better regarded as an option for patients who respond poorly to, or cannot tolerate, corticosteroids, as the authors of one trial suggest [70]. Cell-based products in atopic dermatitis and SSc, and exosome or secretome products in psoriasis and alopecia areata, are best regarded as investigational. These statements describe the pattern of the mapped evidence, not its certainty, because no critical appraisal was done.

Biological Considerations

The patterns above are consistent with the mechanisms proposed for each product group (Figure 3A). Platelet concentrates release growth factors that support repair and modulate inflammation [19,20], MSCs and adipose-derived stem cells act largely through paracrine and immunomodulatory signaling [21], and exosomes carry part of the same signaling in cell-free form [22,51]. The included clinical studies measured these mechanisms only rarely (see Results), so the mechanistic labels in Figure 1 and Figure 3A describe biology proposed in the literature, not findings of this review. Two preclinical or mechanistic records were retained as context: a systematic review and meta-analysis of preclinical MSC studies in autoimmune disease [53] and a scoping review of microRNAs and MSCs in inflammatory and neoplastic skin disease, which concluded that the topic is of growing interest but remains a matter of opinion [54]. Both address biological plausibility, not clinical efficacy in the conditions mapped here.

Gaps in the Clinical Evidence

Five gaps in the clinical evidence stand out. First, product description is poorly documented. One review notes that PRP isolation processes are often poorly described, which limits comparison, reproducibility, and the development of a standard protocol [25], and a recent editorial likewise calls for explicit definitions and characterization of PRP, SVF, MSC, and extracellular vesicle products [47]. Preparation variables (centrifugation, platelet concentration, activation, cell source and dose, vesicle isolation) were not charted here, so how common the problem is across the mapped studies is unknown; they should be charted in future maps. Second, comparators are often weak or absent. Several trials used split-body designs with small samples, and review authors rated the pooled evidence low quality [24,62]; comparator type was not charted systematically. Third, HS, a condition with clear unmet need, had no study identified by the filtered search, and the HS reports found by the supplementary search are limited to one comparative retrospective study, one pilot case series, and five case reports of PRP, PRF, fat, dermal matrix, and allograft [98-104]; adequately designed trials are missing. Fourth, no clinical study of scaffolds, skin substitutes, or bioprinted constructs was identified by the filtered search. The HS dermal matrix, extracellular matrix, and allograft reports above are the only clinical biomaterial use noted, and biomaterial strategies are otherwise discussed in narrative reviews of exosome delivery [23,51]. Fifth, long-term safety and durability data for allogeneic cell products and exosomes were sparse in the sources reviewed. Future trials should be pre-registered, adequately powered, and blinded where possible, report patient-relevant outcomes, and test regenerative products alongside, and not in place of, established therapy.

Limitations

The gaps above describe the clinical evidence; the limitations below describe how this map was built, and some apparent gaps may reflect the search and not the evidence. For example, the absence of HS and biomaterial studies in the filtered search was partly a consequence of the publication-type limit. Only PubMed was searched; hence, relevant studies indexed only in Embase, Scopus, the Cochrane Library, or other sources will have been missed, the reference lists of the included reviews were not screened, and the findings are a map of PubMed-indexed evidence. The search was also restricted by publication type. These labels are assigned by indexers, are applied late to recent records, and exclude case reports, case series, and many retrospective studies. The restriction demonstrably missed HS reports (see Results), and the supplementary search was confined to HS, so comparable reports for other conditions may also have been missed; this probably understates the volume of exploratory work, particularly for exosomes, nanofat, and fat grafting, for which no dedicated search terms were used. Reviews and primary studies were counted together, and because reviews pool the same trials, the counts overstate the amount of independent evidence; overlap was not quantified. A small number of included reviews also covered conditions outside the listed conditions (such as androgenetic alopecia or chronic wounds) or were mainly preclinical, and one included study enrolled healthy volunteers; these are flagged in the notes to Table 2, and the totals would change by one for each record excluded (Appendix D). One reviewer screened records at the abstract level, and the independent check by a second reviewer covered only a 20-record sample (20/20 concordant). Full texts were not reviewed, and several abstracts did not report sample size or results; these are marked in Table 2. Charted figures were re-checked against the PubMed records after charting, and several errors were corrected, which illustrates the error risk of single-reviewer, abstract-based charting. Condition and modality categories are single primary labels, which simplifies mixed studies; borderline assignments are documented in Appendix D. Chronic wounds were excluded by design, and only English-language studies were considered. Consistent with scoping-review methods, no risk-of-bias assessment was undertaken, so the findings describe the extent and character of the evidence and not its certainty.

## Conclusions

Regenerative approaches in these dermatoses have mostly meant PRP used as an adjunct to other treatment or as an alternative to corticosteroids, tested in small, heterogeneous studies. Within the PubMed-indexed evidence mapped here, pooled analyses reported greater repigmentation when PRP was added to light or laser therapy in vitiligo (although several trials found no significant added benefit), and trials in oral lichen planus found no significant difference between PRP or PRF and corticosteroids. MSC-based products have early efficacy reports in atopic dermatitis, while the evidence for psoriasis, lichen sclerosus, and exosome or secretome products is thin. The filtered search found no clinical studies of HS or biomaterial-based strategies, although HS reports exist outside the filter. The main research priorities are standardized reporting of product preparation, adequately powered randomized trials with defined comparators, and multi-database searches without publication-type limits to test how complete this map is.

## Appendices

Appendix A

Search Strategy

PubMed (main search 10 October 2026; supplementary hidradenitis suppurativa search 27 October 2026). The disease block was combined with each modality block and with the limits shown.

Disease block: (psoriasis[tiab] OR "atopic dermatitis"[tiab] OR "alopecia areata"[tiab] OR vitiligo[tiab] OR "lichen planus"[tiab] OR "lichen sclerosus"[tiab] OR "hidradenitis suppurativa"[tiab] OR morphea[tiab] OR "systemic sclerosis"[tiab] OR scleroderma[tiab])

S1 (56 records): ("platelet-rich plasma"[tiab] OR "platelet rich plasma"[tiab] OR "platelet-rich fibrin"[tiab])

S2 (39 records): ("mesenchymal stem cell*"[tiab] OR "mesenchymal stromal cell*"[tiab] OR "stromal vascular fraction"[tiab] OR nanofat[tiab] OR "adipose-derived"[tiab])

S3 (13 records): (exosome*[tiab] OR "extracellular vesicle*"[tiab] OR secretome[tiab])

S4 (5 records): (scaffold*[tiab] OR biomaterial*[tiab] OR bioprinting[tiab] OR "skin substitute*"[tiab])

Limits applied to each main search: 2018/01/01:2026/10/10[dp] AND english[la] AND (clinical trial[pt] OR meta-analysis[pt] OR systematic review[pt] OR observational study[pt])

Supplementary hidradenitis suppurativa search (27 October 2026; no publication-type limit): "hidradenitis suppurativa"[tiab] AND each block S1 to S3 as above, and S4 with "dermal matrix"[tiab] OR "dermal substitute*"[tiab] added to the block; limits 2018/01/01:2026/10/10[dp] AND english[la]. Records: S1, 2; S2, 5; S3, 3; S4, 9 (19 in total; 17 unique).

Appendix B

**Table 3:** PRISMA-ScR checklist PRISMA-ScR: Preferred Reporting Items for Systematic Reviews and Meta-Analyses extension for Scoping Reviews

Item	Checklist element	Reported	Location or note
1	Title: identify the report as a scoping review	Yes	Title
2	Structured summary	Yes	Abstract
3	Rationale	Yes	Introduction
4	Objectives (population, concept, and context question)	Yes	Introduction, final paragraph
5	Protocol and registration	Partial	Registered retrospectively on the Open Science Framework (Methods: Protocol and reporting)
6	Eligibility criteria	Yes	Methods: Eligibility criteria
7	Information sources	Partial	PubMed only (stated as a limitation)
8	Search (full strategy for at least one database)	Yes	Appendix A, including the supplementary hidradenitis suppurativa search
9	Selection of sources of evidence	Partial	One reviewer (S.A.); second reviewer (G.A.) verified a 20-record sample, 20 of 20 concordant
10	Data charting process	Yes	Charted by S.A. from abstracts; figures re-checked against PubMed records by G.A.
11	Data items	Yes	Table 2
12	Critical appraisal of sources (optional)	Not done	Not undertaken
13	Synthesis of results (methods)	Yes	Methods: Data charting and synthesis
14	Selection of sources of evidence (results)	Yes	Figure 2
15	Characteristics of sources of evidence	Yes	Table 1 and Table 2
16	Critical appraisal within sources (optional)	Not done	Not undertaken
17	Results of individual sources of evidence	Yes	Table 2
18	Synthesis of results	Yes	Results by condition and by modality
19	Summary of evidence	Yes	Discussion
20	Limitations	Yes	Discussion: Limitations
21	Conclusions	Yes	Conclusions
22	Funding	Yes	Disclosures

Appendix C

**Table 4:** The screening log (99 records, with the reason for each exclusion)

Record no.	PMID	DOI	First author	Year	Article title	Found in search	Decision	Reason for exclusion	In 20-record verification sample (Y/N)	Second reviewer concordant (Y/N)	Reference no. in the manuscript
1	42596527	10.1177/15473287261475857	Cevallos-Cueva	2026	The potential role of mesenchymal stem cell therapy for moderate-to-severe atopic dermatitis: a systematic review and meta-analysis of human clinical trials	S2	Included	-	N	-	15
2	38878236	10.1007/s00403-024-03035-8	Abdel-Hamid	2024	Effectiveness of fractional erbium-YAG laser, microneedling, platelet-rich plasma in localized stable vitiligo patients: randomized clinical trial	S1	Included	-	N	-	16
3	38324287	10.1001/jamadermatol.2023.5908	Liu	2024	Clinical, histologic, and transcriptomic evaluation of sequential fat grafting for morphea: a nonrandomized controlled trial	S2	Included	-	N	-	17
4	38851685	10.1186/s12903-024-04443-y	Azizi	2024	Comparison of autologous platelet concentrates and topical steroids on oral lichen planus: a systematic review and meta-analysis	S1	Included	-	N	-	18
5	34967722	10.2450/2021.0216-21	Cruciani	2023	Platelet-rich plasma for the treatment of alopecia: a systematic review and meta-analysis	S1	Included	-	N	-	24
6	31211715	10.1097/DSS.0000000000001965	Hesseler	2020	Platelet-rich plasma and its utilities in alopecia: a systematic review	S1	Included	-	N	-	25
7	36507528	10.3389/fmed.2022.1058431	Tejapira	2022	Platelet-rich plasma in alopecia areata and primary cicatricial alopecias: a systematic review	S1	Included	-	N	-	26
8	41564112	10.1111/pcmr.70065	Liu	2026	Excimer 308-nm light in the combination treatment of vitiligo: a systematic review and network meta-analysis	S1	Included	-	N	-	27
9	40386849	10.1111/jocd.70245	Feng	2025	Efficacy and safety of combined platelet-rich plasma with fractional laser for adult patients with vitiligo: a systematic review and meta-analysis of randomized controlled trials	S1	Included	-	N	-	28
10	36062405	10.1177/03000605221119646	Chen	2022	Meta-analysis of the efficacy of adding platelet-rich plasma to 308-nm excimer laser for patients with vitiligo	S1	Included	-	N	-	29
11	33245822	10.1111/iwj.13524	Chen	2021	Current art of combination therapy with autologous platelet-rich plasma for stable vitiligo: a meta-analysis	S1	Included	-	N	-	30
12	40382472	10.1038/s41432-025-01145-4	Sivakumar	2025	Efficacy of intralesional platelet-rich plasma versus intralesional corticosteroids in the management oral lichen planus: a systematic review and meta-analysis of randomized controlled trials using random effects and weighted least square methods	S1	Included	-	N	-	31
13	40437498	10.1186/s12903-025-06189-7	Niemczyk	2025	Use of platelet-rich plasma (PRP) and injectable platelet-rich fibrin (i-PRF) in oral lichen planus treatment: a systematic review of randomized controlled trials	S1	Included	-	N	-	32
14	37109704	10.3390/medicina59040746	Sriram	2023	Efficacy of platelet-rich plasma therapy in oral lichen planus: a systematic review	S1	Included	-	N	-	33
15	37382451	10.55563/clinexprheumatol/ycy3k7	Cao	2023	Autologous fat or adipose-derived stem cell grafting in systemic sclerosis treatment: a systematic review and meta-analysis	S2	Included	-	N	-	34
16	37826898	10.1016/j.semarthrit.2023.152266	Suliman	2023	Surgical management of digital ulcers in systemic sclerosis: a systematic literature review	S2	Included	-	N	-	35
17	38976297	10.55563/clinexprheumatol/y2p4ib	Schipper	2024	Regenerative treatments for scleroderma in cutaneous manifestations of the face: a systematic review	S1, S2, S4	Included	-	N	-	36
18	41718824	10.1007/s10103-026-04799-3	Jafarzadeh	2026	A systematic review of therapeutic procedures for the treatment of morphea and systemic sclerosis	S2	Included	-	N	-	37
19	41508623	10.1142/S2424835526500098	Thakker	2026	Clinical applications of autologous fat grafting in pathological hand conditions	S2	Included	-	N	-	38
20	40014116	10.1007/s00403-025-03934-4	Nilforoushzadeh	2025	Efficacy and safety of platelet-rich plasma therapy in systemic sclerosis and localized scleroderma; a systematic review	S1, S2	Included	-	N	-	39
21	35313985	10.1186/s13287-022-02786-3	Cui	2022	Efficacy and safety of mesenchymal stem cells in the treatment of systemic sclerosis: a systematic review and meta-analysis	S2	Included	-	Y	Y	40
22	33878487	10.1016/j.autrev.2021.102831	Escobar-Soto	2021	Human mesenchymal stem cells for the management of systemic sclerosis. Systematic review	S2	Included	-	N	-	41
23	40054039	10.1016/j.retram.2025.103504	Dey	2025	Efficacy of mesenchymal stem cell transplantation therapy to mitigate psoriasis: a systematic review and meta-analysis	S2	Included	-	Y	Y	42
24	41515899	10.3390/ijms27010021	Bulgaru-Iliescu	2025	Exosome-based therapies for alopecia areata: a systematic review of clinical and experimental evidence	S3	Included	-	Y	Y	43
25	38886861	10.1186/s13287-024-03800-6	Jafarzadeh	2024	Regenerative medicine in the treatment of specific dermatologic disorders: a systematic review of randomized controlled clinical trials	S1, S2	Included	-	N	-	44
26	40918108	10.3389/fimmu.2025.1617157	Matwiejuk	2025	Adipose-derived mesenchymal stem cells and their derivatives in inflammatory skin diseases: a systematic review	S2, S3	Included	-	Y	Y	45
27	31009668	10.1016/j.jaad.2019.04.037	Hesseler	2019	Platelet-rich plasma and its utility in medical dermatology: a systematic review	S1	Included	-	N	-	46
28	39918488	10.1016/j.jcyt.2025.01.007	Chandran	2025	A phase 1, open-label study to determine safety and tolerability of the topical application of mesenchymal stem/stromal cell (MSC) exosome ointment to treat psoriasis in healthy volunteers	S3	Included	-	N	-	52
29	38657832	10.1016/j.biochi.2024.04.009	Swain	2024	Mesenchymal stem cells in autoimmune disease: a systematic review and meta-analysis of pre-clinical studies	S2	Context only	-	N	-	53
30	37239847	10.3390/ijms24108502	Di Vincenzo	2023	miRNAs, mesenchymal stromal cells and major neoplastic and inflammatory skin diseases: a page being written: a systematic review	S2, S3	Context only	-	N	-	54
31	38288773	10.1111/phpp.12944	Omar	2024	A triple combination of latanoprost, fractional COlaser, and platelet-rich plasma in localized vitiligo: a clinical and histopathologic study	S1	Included	-	N	-	55
32	31541597	10.1111/jocd.13103	Khattab	2020	Evaluation of combined excimer laser and platelet-rich plasma for the treatment of nonsegmental vitiligo: a prospective comparative study	S1	Included	-	Y	Y	56
33	34719101	10.1111/jocd.14582	Elsaadany	2022	Comparison between 308-nm excimer light alone versus 308-nm excimer light and platelet-rich plasma in the treatment for localized vitiligo	S1	Included	-	N	-	57
34	33236202	10.1007/s10103-020-03195-9	Afify	2021	Fractional CO2 laser, platelet rich plasma and narrow band ultraviolet B in the treatment of vitiligo (a randomized clinical trial)	S1	Included	-	N	-	58
35	37228170	10.1111/ajd.14080	Abdel Halim	2023	The value of adding platelet-rich plasma (PRP) to noncultured epidermal cell suspension (NCECS) in surgical treatment of stable resistant vitiligo: a self-controlled randomised double-blinded study	S1	Included	-	N	-	59
36	30506679	10.1111/ijd.14286	Parambath	2019	Use of platelet-rich plasma to suspend noncultured epidermal cell suspension improves repigmentation after autologous transplantation in stable vitiligo: a double-blind randomized controlled trial	S1	Included	-	N	-	60
37	37984828	10.1111/phpp.12929	Fawzy	2024	Narrowband ultraviolet B phototherapy combined with intralesional injection of either latanoprost or platelet-rich plasma for stable nonsegmental vitiligo	S1	Included	-	Y	Y	61
38	39509558	10.1111/jocd.16660	Jafarzadeh	2025	A systematic review of case series and clinical trials investigating regenerative medicine for the treatment of vitiligo	S1, S2, S3	Included	-	N	-	62
39	35917262	10.1097/DSS.0000000000003503	McCrary	2022	Utilization of our toolkit: a systematic review and meta-analysis of surgical therapies in vitiligo treatment	S1	Included	-	N	-	63
40	37753680	10.1111/srt.13444	Wang	2023	The use of platelet-rich plasma in wound healing and vitiligo: a systematic review and meta-analysis	S1	Included	-	N	-	64
41	32721141	10.36849/JDD.2020.5192	Juhasz	2020	The utility of platelet-rich plasma for the treatment of alopecia	S1	Included	-	Y	Y	65
42	34014340	10.1007/s00403-021-02238-7	Kang	2022	The safety and efficacy of autologous adipose-derived stromal vascular fraction for nonscarring alopecia: a systematic review	S2	Included	-	N	-	66
43	33421285	10.1111/dth.14768	Roohaninasab	2021	Systematic review of platelet-rich plasma in treating alopecia: focusing on efficacy, safety, and therapeutic durability	S1	Included	-	Y	Y	67
44	31403534	10.1097/DSS.0000000000002053	Marchitto	2019	Emerging nonsteroid-based procedural therapies for alopecia areata: a systematic review	S1	Included	-	Y	Y	68
45	36566395	10.55519/JAMC-04-10933	Khan	2022	Comparative study between intralesional injection of platelet rich plasma and intra lesional triamcinolone for the treatment of alopecia areata	S1	Included	-	N	-	69
46	33034942	10.1111/dth.14388	Hegde	2020	A randomized, placebo and active controlled, split scalp study to evaluate the efficacy of platelet-rich plasma in patchy alopecia areata of the scalp	S1	Included	-	N	-	70
47	38840345	10.29271/jcpsp.2024.06.650	Ghafoor	2024	The role of 5% minoxidil versus platelet-rich plasma in treatment of alopecia areata	S1	Included	-	N	-	71
48	37313640	10.1111/jocd.15805	Abd El-Magid	2023	Diphenylcyclopropenone and platelet-rich plasma in the management of severe or recalcitrant alopecia areata	S1	Included	-	N	-	72
49	33359595	10.1016/j.jaad.2020.12.039	Gupta	2021	Evaluation of platelet-rich plasma on hair regrowth and lesional T-cell cytokine expression in alopecia areata: a randomized observer-blinded, placebo-controlled, split-head pilot study	S1	Included	-	N	-	73
50	33348935	-	Ragab	2020	Platelet-rich plasma in alopecia areata: intradermal injection versus topical application with transepidermal delivery via either fractional carbon dioxide laser or microneedling	S1	Included	-	Y	Y	74
51	39630665	10.36849/jdd.7813	Suh	2024	Evaluating anti-inflammatory potential of platelet-rich plasma in scarring alopecia: a systematic review	S1	Included	-	N	-	75
52	35218680	10.1002/cre2.550	Hijazi	2022	Efficacy of intralesional injections of platelet-rich plasma in patients with oral lichen planus: a pilot randomized clinical trial	S1	Included	-	N	-	76
53	34614123	10.1590/1678-7757-2021-0180	Saglam	2021	Efficacy of injectable platelet-rich fibrin in the erosive oral lichen planus: a split-mouth, randomized, controlled clinical trial	S1	Included	-	N	-	77
54	33415379	10.1007/s00784-020-03702-w	Bennardo	2021	Efficacy of platelet-rich fibrin compared with triamcinolone acetonide as injective therapy in the treatment of symptomatic oral lichen planus: a pilot study	S1	Included	-	Y	Y	78
55	35593522	10.1111/odi.14261	Al-Hallak	2023	Efficacy of injectable platelet-rich fibrin in the treatment of symptomatic oral lichen planus	S1	Included	-	N	-	79
56	39923155	-	Huang	2024	Effectiveness of platelet-rich plasma gel alongside tacrolimus ointment in managing erosive oral lichen planus and its effect on oral immune microenvironment	S1	Included	-	N	-	80
57	41009377	10.3390/ijms26188808	Beutler	2025	Regenerative approaches in vulvar lichen sclerosus: a systematic review	S1, S2	Included	-	N	-	81
58	38079459	10.1097/JU.0000000000003804	Shieh	2024	Conservative management of penile and urethral lichen sclerosus: a systematic review	S1	Included	-	N	-	82
59	41168677	10.1093/jsxmed/qdaf307	De Ponte	2026	Platelet-rich plasma in the management of vulvovaginal disorders: a systematic review	S1	Included	-	N	-	83
60	35048150	10.1007/s00266-021-02718-1	Gutierrez-Ontalvilla	2022	The effect of lipofilling and platelet-rich plasma on patients with moderate-severe vulvar lichen sclerosus who were non-responders to topical clobetasol propionate: a randomized pilot study	S1, S2	Included	-	N	-	84
61	32888384	10.1111/dth.14277	Tedesco	2020	Adipose tissue stromal vascular fraction and adipose tissue stromal vascular fraction plus platelet-rich plasma grafting: new regenerative perspectives in genital lichen sclerosus	S1, S2	Included	-	N	-	85
62	30639885	10.1016/j.jaad.2018.12.060	Goldstein	2019	A randomized double-blind placebo-controlled trial of autologous platelet-rich plasma intradermal injections for the treatment of vulvar lichen sclerosus	S1	Included	-	Y	Y	86
63	35358372	10.1002/art.42133	Khanna	2022	Adipose-derived regenerative cell transplantation for the treatment of hand dysfunction in systemic sclerosis: a randomized clinical trial	S2	Included	-	Y	Y	87
64	34297066	10.1093/rheumatology/keab584	Daumas	2022	Adipose tissue-derived stromal vascular fraction for treating hands of patients with systemic sclerosis: a multicentre randomized trial autologous AD-SVF versus placebo in systemic sclerosis	S2	Included	-	N	-	88
65	37578960	10.1371/journal.pone.0289594	Iglesias	2023	Adipose derived stromal vascular fraction and fat graft for treating the hands of patients with systemic sclerosis. a randomized clinical trial	S2	Included	-	N	-	89
66	33871949	10.1002/sctm.20-0419	Wang	2021	A pilot study on ex vivo expanded autologous adipose-derived stem cells of improving fat retention in localized scleroderma patients	S2	Included	-	N	-	90
67	31314805	10.1371/journal.pone.0218068	Almadori	2019	Stem cell enriched lipotransfer reverses the effects of fibrosis in systemic sclerosis	S2	Included	-	N	-	91
68	35927231	10.1038/s41392-022-01059-y	Cheng	2022	Human umbilical cord mesenchymal stem cells for psoriasis: a phase 1/2a, single-arm study	S2	Included	-	N	-	92
69	40619420	10.1186/s13287-025-04415-1	Elgueta	2025	Novel sponge formulation of mesenchymal stem cell secretome and hyaluronic acid: a safe and effective topical therapy for psoriasis vulgaris	S2, S3	Included	-	N	-	93
70	36499401	10.3390/ijms232315080	Diotallevi	2022	Mesenchymal stem cells and psoriasis: systematic review	S2	Included	-	N	-	94
71	41331725	10.1186/s13287-025-04763-y	Seok	2025	Efficacy and safety of autologous adipose-derived stem cells in subjects with moderate to severe atopic dermatitis: a multicenter, randomized, single-blind, placebo-controlled, phase 2 trial	S2	Included	-	Y	Y	95
72	38944393	10.1016/j.jaci.2024.06.013	Seo	2024	Phase 1/2 trials of human bone marrow-derived clonal mesenchymal stem cells for treatment of adults with moderate to severe atopic dermatitis	S2	Included	-	N	-	96
73	33152947	10.1016/j.biopha.2020.110789	Kim	2020	Effects of conditioned media from human umbilical cord blood-derived mesenchymal stem cells in the skin immune response	S2	Included	-	N	-	97
74	39779541	10.1007/s10103-024-04259-w	Atefi	2025	Evaluation of the efficacy, safety, and satisfaction rate of topical latanoprost in patients with hypopigmented burn scars treated with fractional CO2 laser: a double-blind randomized controlled clinical trial	S1	Excluded	Condition not in scope	N	-	-
75	40777013	10.3389/fimmu.2025.1616231	Chen	2025	Global clinical trials on stem cell therapy for autoimmune diseases: trends and future directions	S2	Excluded	Condition not in scope	N	-	-
76	39086171	10.1111/prd.12582	Davies	2024	Autologous platelet concentrates in esthetic medicine	S1	Excluded	Condition not in scope	N	-	-
77	40414798	10.1111/jocd.70208	Estupiñan	2025	Adipose mesenchymal stem cell-derived exosomes versus platelet-rich plasma treatment for photoaged facial skin: an investigator-blinded, split-face, non-inferiority trial	S1, S2, S3	Excluded	Condition not in scope	N	-	-
78	30606055	10.1177/1203475418818073	Gupta	2019	The efficacy of platelet-rich plasma in the field of hair restoration and facial aesthetics-a systematic review and meta-analysis	S1	Excluded	Condition not in scope	Y	Y	-
79	39279329	10.1111/jocd.16548	Iranmanesh	2024	Comparison of the effectiveness of platelet-rich plasma versus tranexamic acid plus vitamin C mesotherapy in the treatment of periorbital hyperpigmentation: a split-site, randomized clinical trial	S1	Excluded	Condition not in scope	N	-	-
80	41748500	10.1111/jocd.70756	Jeong	2026	A split-scar study: study of surgical scar using exosomes	S3	Excluded	Condition not in scope	N	-	-
81	32107629	10.1007/s00403-020-02049-2	Liu	2020	Efficacy of a wound-dressing biomaterial on prevention of postinflammatory hyperpigmentation after suction blister epidermal grafting in stable vitiligo patients: a controlled assessor-blinded clinical study with in vitro bioactivity investigation	S4	Excluded	Condition not in scope	N	-	-
82	34359858	10.3390/cells10071688	Tura-Ceide	2021	Circulating cell biomarkers in pulmonary arterial hypertension: relationship with clinical heterogeneity and therapeutic response	S3	Excluded	Condition not in scope	N	-	-
83	38970896	10.1016/j.semarthrit.2024.152498	Zeng	2024	Efficacy and safety of culture-expanded mesenchymal stromal cell therapy in the treatment of 4 types of inflammatory arthritis: a systematic review and meta-analysis of 36 randomized controlled trials	S2	Excluded	Condition not in scope	N	-	-
84	39934871	10.1186/s13287-025-04184-x	Zeng	2025	Efficacy and safety of mesenchymal stromal cell transplantation in the treatment of autoimmune and rheumatic immune diseases: a systematic review and meta-analysis of randomized controlled trials	S2	Excluded	Condition not in scope	N	-	-
85	33210178	10.1007/s00011-020-01412-3	Campanati	2020	The efficacy of in vivo administration of apremilast on mesenchymal stem cells derived from psoriatic patients	S2	Excluded	Intervention not regenerative	Y	Y	-
86	41730404	10.1016/j.autrev.2026.104009	Gkouvi	2026	Hyaluronic acid fillers in systemic sclerosis and localized scleroderma: a systematic review	S1	Excluded	Intervention not regenerative	Y	Y	-
87	32712160	10.1016/j.jid.2020.07.009	Jiang	2020	Small extracellular vesicles containing miR-381-3p from keratinocytes promote T helper type 1 and T helper type 17 polarization in psoriasis	S3	Excluded	Intervention not regenerative	N	-	-
88	39985455	10.1093/rheumatology/keaf109	Kaufman	2025	Avenciguat: a novel soluble guanylate cyclase activator that affects multiple cell types to inhibit IFN-1 signalling and fibrosis	S1	Excluded	Intervention not regenerative	N	-	-
89	30513014	10.1080/09546634.2018.1544405	Rinaldi	2019	Randomized controlled trial on a PRP-like cosmetic, biomimetic peptides based, for the treatment of alopecia areata	S1	Excluded	Intervention not regenerative	Y	Y	-
90	40278948	10.1007/s00403-025-04191-1	Wang	2025	Hematopoietic stem cell translation for relapse of psoriasis. Systematic review	S2	Excluded	Intervention not regenerative	N	-	-
91	39151120	10.1021/acs.jmedchem.4c01305	Zheng	2024	Discovery of KT-474─a potent, selective, and orally bioavailable IRAK4 degrader for the treatment of autoimmune diseases	S4	Excluded	Intervention not regenerative	N	-	-
92	40888924	10.1007/s00296-025-05953-w	Chatzopoulos	2025	Small extracellular vesicle cargo as biomarkers in autoimmune rheumatic diseases: a systematic review	S3	Excluded	Non-interventional or protocol	N	-	-
93	31465725	-	Chen	2019	Novel findings from determination of common expressed plasma exosomal microRNAs in patients with psoriatic arthritis, psoriasis vulgaris, rheumatoid arthritis, and gouty arthritis	S3	Excluded	Non-interventional or protocol	Y	Y	-
94	30457999	10.1371/journal.pone.0206545	Hoffmann-Vold	2018	Augmented concentrations of CX3CL1 are associated with interstitial lung disease in systemic sclerosis	S4	Excluded	Non-interventional or protocol	N	-	-
95	32147195	10.1016/j.jdermsci.2020.02.009	Li	2020	The profiles of miRNAs and lncRNAs in peripheral blood neutrophils exosomes of diffuse cutaneous systemic sclerosis	S3	Excluded	Non-interventional or protocol	N	-	-
96	30428931	10.1186/s13287-018-1053-4	Liang	2018	Safety analysis in patients with autoimmune disease receiving allogeneic mesenchymal stem cells infusion: a long-term retrospective study	S2	Excluded	Non-interventional or protocol	Y	Y	-
97	38857576	10.1159/000538993	Ubago-Rodríguez	2024	Challenges in psoriasis research: a systematic review of preclinical models	S4	Excluded	Non-interventional or protocol	N	-	-
98	30127049	10.1136/bmjopen-2017-020479	van Rhijn-Brouwer	2018	A randomised placebo-controlled double-blind trial to assess the safety of intramuscular administration of allogeneic mesenchymal stromal cells for digital ulcers in systemic sclerosis: the MANUS Trial protocol	S2	Excluded	Non-interventional or protocol	N	-	-
99	28834191	10.1111/jocd.12397	Abdelghani	2017	Combined treatment with fractional carbon dioxide laser, autologous platelet-rich plasma, and narrow band ultraviolet B for vitiligo in different body sites: a prospective, randomized comparative trial	S1	Excluded	Outside date window	N	-	-

Appendix D

**Table 5:** Each row is one included source, listed in order of reference number. Columns give the order, reference number, first author, year, journal, DOI, primary condition label, primary modality label, source type (review or primary study), design, number of studies included, number of participants, principal finding as stated in the abstract, and a flag for borderline or limited records (a dash means no flag). The Excel workbook also contains the category-assignment rules, a recomputation of Table 1 from the dataset, the supplementary hidradenitis suppurativa records, and the search log EASI: Eczema Area and Severity Index; LoSDI: Localized Scleroderma Skin Damage Index; mLoSSI: modified Localized Scleroderma Severity Index; PRF: platelet-rich fibrin; PRP: platelet-rich plasma; SMD: standardized mean difference; SVF: stromal vascular fraction; REU: reticulation/erythema/ulceration

Order	Reference no.	First author	Year	Journal	DOI	Condition (primary label)	Modality (primary label)	Source type	Design	Studies included	Participants	Principal finding (as stated in abstract)	Flag (borderline or limited record)
1	15	Cevallos-Cueva	2026	Stem Cells Dev	10.1177/15473287261475857	Atopic dermatitis	Cell-based	Review	Systematic review and meta-analysis (4 of 5 trials randomized)	5	236	Pooled EASI-50 response proportion at week 12 46.76% (95% CI 32.36 to 61.72) and pooled EASI-75 proportion at week 16 23.97% (95% CI 16.48 to 33.50); single-group response proportions, not comparisons with placebo. Treatment-emergent adverse events 26.86% (95% CI 19.56 to 35.68)	-
2	16	Abdel-Hamid	2024	Arch Dermatol Res	10.1007/s00403-024-03035-8	Vitiligo	PRP or PRF	Primary study	Randomized controlled trial	Not applicable	40	Erbium:YAG laser plus PRP and microneedling plus PRP both repigmented (20 per arm; no non-PRP control); side effects 5% versus 25%	No non-PRP control
3	17	Liu	2024	JAMA Dermatol	10.1001/jamadermatol.2023.5908	Systemic sclerosis or morphea	Cell-based	Primary study	Non-randomized controlled trial	Not applicable	44	Sequential fat grafting with SVF gel versus fat grafting alone (morphea): 12-month decrease in LoSDI 4.3 versus 2.1 (p<0.001); decrease in mLoSSI 1.6 versus 0.9 (p=0.13); histologic and transcriptomic antifibrotic signals	-
4	18	Azizi	2024	BMC Oral Health	10.1186/s12903-024-04443-y	Oral lichen planus	PRP or PRF	Review	Systematic review and meta-analysis	6	109	Platelet concentrates versus topical steroids: pain SMD 0.17 (95% CI -0.47 to 0.81), no significant difference; Thongprasom score SMD -2.88 (95% CI -5.51 to -0.25), described by the authors as not significant although the interval excludes zero	-
5	24	Cruciani	2023	Blood Transfus	10.2450/2021.0216-21	Alopecia	PRP or PRF	Review	Systematic review and meta-analysis	27	1,117	Controlled trials (18 in androgenetic alopecia, 9 in alopecia areata): hair density mean difference 25.6 hairs/cm^2^ (95% CI 2.62 to 48.57) versus saline in androgenetic alopecia, rated low quality by the authors; alopecia areata evidence (PRP versus triamcinolone) rated very low quality	Out-of-scope condition (androgenetic alopecia)
6	25	Hesseler	2020	Dermatol Surg	10.1097/DSS.0000000000001965	Alopecia	PRP or PRF	Review	Systematic review	19	460	Articles covered alopecia areata (3 studies, 71 patients) and androgenetic alopecia (16 studies, 389 patients); most studies reported hair growth; heterogeneity prevented pooling	Out-of-scope condition (androgenetic alopecia)
7	26	Tejapira	2022	Front Med (Lausanne)	10.3389/fmed.2022.1058431	Alopecia	PRP or PRF	Review	Systematic review	32	621	Participants had alopecia areata (a further 19 had primary cicatricial alopecia); PRP superior as monotherapy in 5 studies and comparable to intralesional steroids in 6 (alopecia areata); alleviated progression in 9 (primary cicatricial alopecia)	Out-of-scope condition (primary cicatricial alopecia)
8	27	Liu	2026	Pigment Cell Melanoma Res	10.1111/pcmr.70065	Vitiligo	PRP or PRF	Review	Network meta-analysis of randomized trials (whole excimer-combination network)	38	1,841	PRP combined with excimer light enhanced 50% or greater repigmentation (risk ratio 1.76; 95% CI 1.32 to 2.35); studies and participants refer to the whole network	-
9	28	Feng	2025	J Cosmet Dermatol	10.1111/jocd.70245	Vitiligo	PRP or PRF	Review	Systematic review and meta-analysis	7	366	PRP plus fractional laser: mean repigmentation grade, mean difference 1.58 (95% CI 1.08 to 2.07; p<0.01); adverse events risk ratio 0.86 (95% CI 0.69 to 1.07; p=0.17)	-
10	29	Chen	2022	J Int Med Res	10.1177/03000605221119646	Vitiligo	PRP or PRF	Review	Meta-analysis	6	302	PRP plus 308-nm excimer improved response versus phototherapy alone; adverse events reported as comparable	-
11	30	Chen	2021	Int Wound J	10.1111/iwj.13524	Vitiligo	PRP or PRF	Review	Systematic review and meta-analysis	11	670	PRP plus excimer or fractional carbon dioxide laser effective; no added benefit reported with narrowband ultraviolet B or noncultured epidermal cell suspension (participants reported as cases)	-
12	31	Sivakumar	2025	Evid Based Dent	10.1038/s41432-025-01145-4	Oral lichen planus	PRP or PRF	Review	Systematic review and meta-analysis (6 of 7 articles pooled)	7	141	PRP versus intralesional steroids: pain effect size -0.34 (95% CI -0.94 to 0.27; p=0.274), lesion size effect size -0.10 (95% CI -0.58 to 0.39; p=0.697); no significant differences; participants are those in the six pooled articles	-
13	32	Niemczyk	2025	BMC Oral Health	10.1186/s12903-025-06189-7	Oral lichen planus	PRP or PRF	Review	Systematic review of randomized controlled trials	7	Not stated	PRP and injectable PRF improved pain and lesion severity; mostly no significant difference versus corticosteroids	-
14	33	Sriram	2023	Medicina (Kaunas)	10.3390/medicina59040746	Oral lichen planus	PRP or PRF	Review	Systematic review	5	Not stated	PRP improved symptoms; efficacy described by the review authors as comparable to corticosteroids	-
15	34	Cao	2023	Clin Exp Rheumatol	10.55563/clinexprheumatol/ycy3k7	Systemic sclerosis or morphea	Cell-based	Review	Systematic review and meta-analysis	15	Not stated	Fingertip symptoms improved; skin thickness change not significant; SVF had highest adverse-event proportion	-
16	35	Suliman	2023	Semin Arthritis Rheum	10.1016/j.semarthrit.2023.152266	Systemic sclerosis or morphea	Cell-based	Review	Systematic review	13	Not stated	Systemic sclerosis digital ulcers: healing 66-100% with fat grafting (4 studies) and 32-60% with SVF (3 studies)	-
17	36	Schipper	2024	Clin Exp Rheumatol	10.55563/clinexprheumatol/y2p4ib	Systemic sclerosis or morphea	Mixed	Review	Systematic review	18	Not stated	Facial scleroderma: most studies showed symptom improvement; no treatment superior; quality weak (15 studies) or moderate (3 studies) as judged by the review authors	-
18	37	Jafarzadeh	2026	Lasers Med Sci	10.1007/s10103-026-04799-3	Systemic sclerosis or morphea	Mixed	Review	Systematic review	28	732	15 of the 28 articles concerned energy-based devices; positive outcomes in all but one intralesional-injection study (SVF gel, fat, PRP plus SVF); no life-threatening adverse events	Broader intervention scope (energy-based devices)
19	38	Thakker	2026	J Hand Surg Asian Pac Vol	10.1142/S2424835526500098	Systemic sclerosis or morphea	Cell-based	Review	Systematic review	25	909	Systemic sclerosis accounted for 7 studies; autologous fat grafting improved pain and function in systemic sclerosis and thumb-base osteoarthritis; heterogeneous techniques	Out-of-scope condition (thumb-base osteoarthritis and other hand conditions)
20	39	Nilforoushzadeh	2025	Arch Dermatol Res	10.1007/s00403-025-03934-4	Systemic sclerosis or morphea	PRP or PRF	Review	Systematic review	Not stated	Not stated	Systemic sclerosis and localized scleroderma: improved mouth handicap, skin elasticity, ulcer healing, and Localized Scleroderma Cutaneous Assessment Tool; adverse events mild and self-limited	-
21	40	Cui	2022	Stem Cell Res Ther	-	Systemic sclerosis or morphea	Cell-based	Review	Systematic review and meta-analysis	9	133	Effects expressed versus baseline; mesenchymal stem or stromal cell therapy improved skin thickening, lung function, mouth opening, and digital ulcers; no severe adverse events	-
22	41	Escobar-Soto	2021	Autoimmun Rev	10.1016/j.autrev.2021.102831	Systemic sclerosis or morphea	Cell-based	Review	Systematic review	11	101	Improvements in vascular and pain outcomes in almost all cases; evidence level mostly 4	-
23	42	Dey	2025	Curr Res Transl Med	10.1016/j.retram.2025.103504	Psoriasis	Cell-based	Review	Systematic review and meta-analysis	21	Not stated	5 clinical and 16 preclinical studies; meta-analysis (10 preclinical studies, 343 animals) showed reduced Psoriasis Area and Severity Index and epidermal thickness; clinical data not pooled	Preclinical component (meta-analysis pooled animal data only)
24	43	Bulgaru-Iliescu	2025	Int J Mol Sci	10.3390/ijms27010021	Alopecia	Exosome or secretome	Review	Systematic review (clinical and experimental evidence)	40	Not stated	Only 2 studies were clinical; density gains 9-31 hairs/cm^2^ in clinical reports; mild transient adverse events; randomized trials lacking	Preclinical or in vitro component
25	44	Jafarzadeh	2024	Stem Cell Res Ther	10.1186/s13287-024-03800-6	Several dermatoses	Mixed	Review	Systematic review	64	2,888	Regenerative methods (PRP, SVF, mesenchymal stem cells, conditioned media, cell transplants) promising across selected dermatoses	Multi-condition review
26	45	Matwiejuk	2025	Front Immunol	10.3389/fimmu.2025.1617157	Several dermatoses	Mixed	Review	Systematic review (preclinical and clinical)	Not stated	Not stated	Psoriasis, atopic dermatitis, localized scleroderma, and acne; adipose-derived mesenchymal stem cells reduced psoriatic features; their exosomes alleviated atopic dermatitis (largely preclinical)	Multi-condition review; preclinical component
27	46	Hesseler	2019	J Am Acad Dermatol	10.1016/j.jaad.2019.04.037	Several dermatoses	PRP or PRF	Review	Systematic review	14	Not stated	Articles covered chronic wounds and vitiligo; two studies documented benefit of adjunctive PRP in stable vitiligo	Out-of-scope condition (chronic wounds); borderline condition label (scope stated as medical dermatology)
28	52	Chandran	2025	Cytotherapy	10.1016/j.jcyt.2025.01.007	Psoriasis	Exosome or secretome	Primary study	Phase 1 safety study	Not applicable	10	Topical mesenchymal stem cell exosome ointment in healthy volunteers: well tolerated; no serious adverse events; no patients with psoriasis enrolled	Healthy volunteers
29	55	Omar	2024	Photodermatol Photoimmunol Photomed	10.1111/phpp.12944	Vitiligo	PRP or PRF	Primary study	Randomized controlled trial	Not applicable	60	Latanoprost plus fractional carbon dioxide laser plus PRP gave greater improvement than latanoprost plus laser or latanoprost alone (three arms)	-
30	56	Khattab	2020	J Cosmet Dermatol	10.1111/jocd.13103	Vitiligo	PRP or PRF	Primary study	Comparative study (indexed as randomized controlled trial)	Not applicable	52	PRP plus excimer produced higher response than excimer alone	-
31	57	Elsaadany	2022	J Cosmet Dermatol	10.1111/jocd.14582	Vitiligo	PRP or PRF	Primary study	Randomized controlled trial	Not applicable	36	Excimer with or without PRP: no significant difference in repigmentation (72 patches)	-
32	58	Afify	2021	Lasers Med Sci	10.1007/s10103-020-03195-9	Vitiligo	PRP or PRF	Primary study	Randomized controlled trial	Not applicable	20	All arms improved; no significant difference between fractional carbon dioxide laser, PRP, and narrowband ultraviolet B combinations	-
33	59	Abdel Halim	2023	Australas J Dermatol	10.1111/ajd.14080	Vitiligo	PRP or PRF	Primary study	Randomized controlled trial	Not applicable	15	Adding PRP to noncultured epidermal cell suspension gave no significant additional benefit in acral or juxta-articular vitiligo (30 lesions)	-
34	60	Parambath	2019	Int J Dermatol	10.1111/ijd.14286	Vitiligo	PRP or PRF	Primary study	Randomized controlled trial	Not applicable	21	Epidermal cell suspension in PRP versus phosphate-buffered saline (two patches per patient): repigmentation 75.6% versus 65% at 6 months (p=0.0036)	-
35	61	Fawzy	2024	Photodermatol Photoimmunol Photomed	10.1111/phpp.12929	Vitiligo	PRP or PRF	Primary study	Randomized controlled trial	Not applicable	60	Narrowband ultraviolet B plus PRP versus plus latanoprost: excellent response 13.3% versus 26.7% (no significant difference in degree of repigmentation); Vitiligo Extent Score for a Target Area favored latanoprost (p=0.032); PRP response described by the authors as more stable	-
36	62	Jafarzadeh	2025	J Cosmet Dermatol	10.1111/jocd.16660	Vitiligo	Mixed	Review	Systematic review	48	2,186	Regenerative approaches reported effective with no serious adverse events; the review authors judged 48.8% of the included studies at high risk of bias	-
37	63	McCrary	2022	Dermatol Surg	10.1097/DSS.0000000000003503	Vitiligo	PRP or PRF	Review	Systematic review and meta-analysis	73	2,911	Surgical therapies reviewed, including PRP; benefit reported for ablative laser or microneedling with narrowband ultraviolet B	Broader intervention scope
38	64	Wang	2023	Skin Res Technol	10.1111/srt.13444	Vitiligo	PRP or PRF	Review	Systematic review and meta-analysis	27	Not stated	Studies covered wounds and vitiligo; PRP gave better repigmentation than controls in vitiligo; wound outcomes outside scope	Out-of-scope condition (chronic wounds)
39	65	Juhasz	2020	J Drugs Dermatol	10.36849/JDD.2020.5192	Alopecia	PRP or PRF	Review	Systematic review	61	Not stated	Preliminary results described as promising for androgenetic alopecia, alopecia areata, and cicatricial alopecia; heterogeneous data	Out-of-scope condition (androgenetic and cicatricial alopecia)
40	66	Kang	2022	Arch Dermatol Res	10.1007/s00403-021-02238-7	Alopecia	Cell-based	Review	Systematic review	6	188	All studies reported improved hair density; no severe adverse events	Out-of-scope condition (androgenetic alopecia)
41	67	Roohaninasab	2021	Dermatol Ther	10.1111/dth.14768	Alopecia	PRP or PRF	Review	Systematic review	8	Not stated	PRP plus minoxidil gave highest improvement; alopecia areata efficacy 31.7-76%	-
42	68	Marchitto	2019	Dermatol Surg	10.1097/DSS.0000000000002053	Alopecia	PRP or PRF	Review	Systematic review (PRP, ultraviolet radiation and laser therapies)	46	Not stated	Of 644 articles screened, 46 were included; strong potential reported but high-quality evidence lacking (authors' statement)	Broader intervention scope
43	69	Khan	2022	J Ayub Med Coll Abbottabad	10.55519/JAMC-04-10933	Alopecia	PRP or PRF	Primary study	Randomized controlled trial	Not applicable	60	Intralesional triamcinolone more effective than PRP for complete response (the abstract states both p=0.05 and p<0.05); 30 per arm	-
44	70	Hegde	2020	Dermatol Ther	10.1111/dth.14388	Alopecia	PRP or PRF	Primary study	Randomized controlled trial (split-scalp)	Not applicable	50	Regrowth greatest with triamcinolone, then PRP, and then placebo (p=0.016 across three arms; no pairwise test in abstract); dermoscopic improvement similar for PRP and triamcinolone (p=0.448)	-
45	71	Ghafoor	2024	J Coll Physicians Surg Pak	10.29271/jcpsp.2024.06.650	Alopecia	PRP or PRF	Primary study	Randomized controlled trial	Not applicable	376	Number as stated in the abstract. PRP versus 5% minoxidil: mean Severity of Alopecia Tool score 1.48 versus 1.54 at 3 months; no significant difference (p=0.483)	-
46	72	Abd El-Magid	2023	J Cosmet Dermatol	10.1111/jocd.15805	Alopecia	PRP or PRF	Primary study	Randomized controlled trial	Not applicable	24	Diphenylcyclopropenone with or without PRP (13 versus 11 patients): regrowth 53.85% versus 54.5%; no significant difference	-
47	73	Gupta	2021	J Am Acad Dermatol	10.1016/j.jaad.2020.12.039	Alopecia	PRP or PRF	Primary study	Randomized controlled trial	Not applicable	27	Severity of Alopecia Tool change not significant versus baseline; interferon-gamma and interleukin-17 messenger RNA fell; interleukin-10 and forkhead box P3 (FOXP3) rose	-
48	74	Ragab	2020	Acta Dermatovenerol Alp Pannonica Adriat	-	Alopecia	PRP or PRF	Primary study	Randomized controlled trial	Not applicable	60	Injection versus laser-assisted or microneedling-assisted topical PRP (three arms): similar improvement; injection more painful	-
49	75	Suh	2024	J Drugs Dermatol	10.36849/jdd.7813	Alopecia	PRP or PRF	Review	Systematic review	11	Not stated	Scarring alopecia: PRP described as slowing progression with reduced inflammatory signs; evidence limited to case reports	Out-of-scope condition (scarring alopecia only; borderline)
50	76	Hijazi	2022	Clin Exp Dent Res	10.1002/cre2.550	Oral lichen planus	PRP or PRF	Primary study	Randomized controlled trial (pilot)	Not applicable	20	PRP versus triamcinolone: complete remission 80% versus 70% (not significant)	-
51	77	Saglam	2021	J Appl Oral Sci	10.1590/1678-7757-2021-0180	Oral lichen planus	PRP or PRF	Primary study	Randomized controlled trial (split-mouth)	Not applicable	24	Injectable PRF versus corticosteroid: similar reductions in pain and lesion size at 6 months	-
52	78	Bennardo	2021	Clin Oral Investig	10.1007/s00784-020-03702-w	Oral lichen planus	PRP or PRF	Primary study	Randomized controlled trial (split-mouth)	Not applicable	Not reported in abstract	PRF versus triamcinolone: lesion reduction 59.8% versus 59.2%; no significant difference	Sample size not reported in abstract
53	79	Al-Hallak	2023	Oral Dis	10.1111/odi.14261	Oral lichen planus	PRP or PRF	Primary study	Randomized controlled trial (split-mouth)	Not applicable	12	Injectable PRF versus triamcinolone at 4 weeks: visual analog scale reduction 68.5% versus 91% and REU score reduction 74% versus 91%; no significant between-group difference (p>0.05; exact value not given in abstract)	-
54	80	Huang	2024	Pak J Pharm Sci	-	Oral lichen planus	PRP or PRF	Primary study	Randomized controlled trial	Not applicable	80	PRP gel plus tacrolimus: higher effective rate, smaller lesion area, lower pain, and lower recurrence than tacrolimus alone	-
55	81	Beutler	2025	Int J Mol Sci	10.3390/ijms26188808	Lichen sclerosus	Mixed	Review	Systematic review	13	Not stated	Studies of PRP, adipose-derived stem cells, and mesotherapy: improved symptoms, quality of life, sexual function, and tissue quality; small samples, few controls, short follow-up	Broader intervention scope (mesotherapy)
56	82	Shieh	2024	J Urol	10.1097/JU.0000000000003804	Lichen sclerosus	PRP or PRF	Review	Systematic review	17	Not stated	Male genital lichen sclerosus: steroids remain mainstay; PRP and tacrolimus supported as alternatives or adjuvants	Broader intervention scope (conservative management)
57	83	De Ponte	2026	J Sex Med	10.1093/jsxmed/qdaf307	Lichen sclerosus	PRP or PRF	Review	Systematic review	18	Not stated	Searched PubMed and Embase to October 2024; PRP injection to the vulvovaginal area as a stand-alone technique; vulvar lichen sclerosus among indications; protocols heterogeneous	Out-of-scope condition (other vulvovaginal disorders)
58	84	Gutierrez-Ontalvilla	2022	Aesthetic Plast Surg	-	Lichen sclerosus	Mixed	Primary study	Randomized controlled trial (pilot)	Not applicable	20	19 assessed. Lipofilling plus PRP versus clobetasol: symptoms, signs, and inflammation improved; no elasticity gain; no related adverse events	-
59	85	Tedesco	2020	Dermatol Ther	10.1111/dth.14277	Lichen sclerosus	Mixed	Primary study	Randomized controlled trial	Not applicable	40	Adipose-derived SVF alone and with PRP both improved at 6 months; combination favored in early disease and discouraged in late disease	-
60	86	Goldstein	2019	J Am Acad Dermatol	10.1016/j.jaad.2018.12.060	Lichen sclerosus	PRP or PRF	Primary study	Randomized controlled trial (letter)	Not applicable	Not reported in abstract	Double-blind placebo-controlled trial of intradermal PRP in vulvar lichen sclerosus; no abstract; outcomes not extractable from the PubMed record	No abstract; outcomes not extractable
61	87	Khanna	2022	Arthritis Rheumatol	10.1002/art.42133	Systemic sclerosis or morphea	Cell-based	Primary study	Randomized controlled trial	Not applicable	88	Adipose-derived regenerative cells (n=48) versus placebo (n=40): primary Cochin Hand Function Scale end point not met (mean improvement at 48 weeks 11.0 versus 8.9; p=0.299); trend in diffuse systemic sclerosis (nominal p=0.069); well tolerated	-
62	88	Daumas	2022	Rheumatology (Oxford)	10.1093/rheumatology/keab584	Systemic sclerosis or morphea	Cell-based	Primary study	Randomized controlled trial (phase 2)	Not applicable	40	Adipose-derived SVF not superior to placebo for hand function at 3 months	-
63	89	Iglesias	2023	PLoS One	10.1371/journal.pone.0289594	Systemic sclerosis or morphea	Cell-based	Primary study	Randomized controlled trial	Not applicable	20	Open label, control medical treatment only; SVF plus fat micrografts: less pain and fewer digital ulcers versus control; no adverse events	-
64	90	Wang	2021	Stem Cells Transl Med	10.1002/sctm.20-0419	Systemic sclerosis or morphea	Cell-based	Primary study	Randomized controlled trial (pilot)	Not applicable	18	Localized scleroderma: adipose-derived stem cell-assisted fat grafting, fat retention 49.8% versus 31.8% (SVF) versus 21.9% (conventional)	-
65	91	Almadori	2019	PLoS One	10.1371/journal.pone.0218068	Systemic sclerosis or morphea	Cell-based	Primary study	Open cohort with in vitro study	Not applicable	62	Oro-facial systemic sclerosis: stem cell-enriched lipotransfer improved fibrosis; reduced transforming growth factor-beta 1 and connective tissue growth factor in co-culture	Preclinical or in vitro component
66	92	Cheng	2022	Signal Transduct Target Ther	10.1038/s41392-022-01059-y	Psoriasis	Cell-based	Primary study	Phase 1/2a single-arm trial	Not applicable	17	Umbilical cord mesenchymal stem cell transplantation safe and partially effective: 8 of 17 (47.1%) had at least 40% Psoriasis Area and Severity Index improvement (2 of 8 men and 6 of 9 women); regulatory T-cell rise and T helper 17 fall in responders	-
67	93	Elgueta	2025	Stem Cell Res Ther	10.1186/s13287-025-04415-1	Psoriasis	Exosome or secretome	Primary study	Randomized controlled trial (indexed)	Not applicable	12	12 patients for efficacy, 7 for safety; secretome with hyaluronic acid sponge: modified Psoriasis Area and Severity Index reduction up to 33%; plaque size down 41%; no adverse reactions	-
68	94	Diotallevi	2022	Int J Mol Sci	10.3390/ijms232315080	Psoriasis	Cell-based	Review	Systematic review (descriptive)	Not stated	Not stated	Describes mesenchymal stem cell interaction with the psoriatic inflammatory microenvironment and therapeutic potential	Descriptive review; no extractable outcome data
69	95	Seok	2025	Stem Cell Res Ther	10.1186/s13287-025-04763-y	Atopic dermatitis	Cell-based	Primary study	Randomized controlled trial (phase 2)	Not applicable	114	Autologous adipose-derived mesenchymal stem cells: EASI-75 at week 16 in 23.7% (14/59) versus 7.3% (4/55) with placebo (p=0.016)	-
70	96	Seo	2024	J Allergy Clin Immunol	10.1016/j.jaci.2024.06.013	Atopic dermatitis	Cell-based	Primary study	Phase 1/2 trial	Not applicable	92	Phase 1 n=20 and phase 2 n=72; clonal bone marrow-derived mesenchymal stem cells: higher EASI-50 response at week 12 versus placebo (p=0.038); Dermatology Life Quality Index and pruritus differences not significant	-
71	97	Kim	2020	Biomed Pharmacother	10.1016/j.biopha.2020.110789	Atopic dermatitis	Exosome or secretome	Primary study	Clinical and in vitro study	Not applicable	Not stated	Mild atopic dermatitis; conditioned medium applied for 4 weeks improved skin barrier (corneometry up, transepidermal water loss down)	Preclinical or in vitro component
